# From Gene to Enzyme: Multidimensional Decoding of the GGT Molecular Family and Its Clinical Tumor Diagnosis

**DOI:** 10.1002/cam4.71165

**Published:** 2025-11-28

**Authors:** Fei Wang, Jianshan Yang, Feng Zhu, Xuebing Xu, Junpeng Zhao, Xudong Xie, Xuyang He, Yuxuan Huang, Lirong Zhou, Xiaogang Hu, Xiaomin Lu, Mingbing Xiao

**Affiliations:** ^1^ Department of Gastroenterology and Medical Laboratory Science Affiliated Hospital of Nantong University, Medical school of Nantong University Nantong Jiangsu China; ^2^ The Armed Police Corps Hospital of Jiangsu Province Yangzhou Jiangsu China; ^3^ Rudong County People's Hospital Nantong Jiangsu China; ^4^ Clinical Medicine Medical School of Nantong University Nantong Jiangsu China; ^5^ Rudong County Hospital of Traditional Chinese Medicine Nantong Jiangsu China; ^6^ Department of Oncology Affiliated Haian Hospital of Nantong University Haian Jiangsu China

**Keywords:** biomarker, GGT enzyme, GGT fraction, GGT gene family, GGT isozyme, GGT mRNA, tumors

## Abstract

**Background:**

Gamma‐glutamyltransferase (GGT) is a membrane‐bound enzyme involved in glutathione metabolism and oxidative stress regulation. Although it is traditionally viewed as a liver function marker, emerging evidence suggests that its aberrant expression is closely associated with tumorigenesis, progression, and therapeutic resistance across multiple solid tumors. However, the comprehensive landscape of the GGT gene family and its clinical value in tumor diagnosis and prognosis remain unclear.

**Objective:**

To systematically review the multidimensional roles of the GGT molecular family—including gene variants, mRNA isoforms, enzyme activity, and protein isoforms—in tumor biology and clinical oncology and to evaluate their potential as diagnostic and prognostic biomarkers.

**Methods:**

We conducted a comprehensive literature review (PubMed, CNKI; inception–August 2025) focusing on (1) GGT family gene structure, expression patterns, and regulatory mechanisms; (2) GGT mRNA splice variants and isoforms; (3) GGT enzymatic activity and posttranslational modifications; and (4) clinical studies evaluating GGT as a biomarker in solid tumors. Data were synthesized narratively, emphasizing molecular mechanisms and clinical significance.

**Results:**

The human GGT family comprises 13 homologous genes (e.g., GGT1‐7 and GGTLC1‐3) localized on chromosomes 20 and 22, which exhibit tissue‐specific expression and functional diversity. GGT1 (22q11.23), which is the most extensively studied gene, is highly expressed in renal cell carcinoma (RCC), hepatocellular carcinoma (HCC), gastric cancer (GC), and breast cancer (BRC) and is correlated with poor prognosis and metastasis. GGT5 acts as a tumor suppressor in HCC but promotes progression in gastric cancer via PI3K/AKT pathway activation. GGT7 overexpression predicts poor survival in patients with HCC and glioblastoma. The GGT‐II isoform demonstrated 78.7% sensitivity and 92.3% specificity for HCC diagnosis, outperforming AFP (AUC: 0.89 vs. 0.67). Serum GGT activity ≥ 50 U/L independently predicts poor overall survival (OS) in patients with HCC (HR: 1.78, 95% CI: 1.26–2.50). GGT mRNA splice variants (e.g., the GGT‐I mRNA‐B subtype) enhance early HCC detection when combined with AFP (sensitivity: 98%).

**Conclusions:**

The GGT molecular family plays pleiotropic roles in tumor biology via redox homeostasis, EMT, and immune modulation. The GGT1/5/7 and GGT‐II isoforms represent promising biomarkers for early diagnosis, prognosis, and therapeutic targeting in multiple cancers. Future multicenter studies should validate GGT‐based biomarker panels and elucidate the mechanisms underlying tissue‐specific GGT functions.

**Abbreviations**
AFP: alpha‐fetoprotein
AFR: albumin/fibrinogen ratio
AFU: α‐L‐fucosidase
ALP: alkaline phosphatase
AST: aspartate aminotransferase
BLC: bladder cancer
BCL: B‐cell lymphoma
Bcl‐2: B‐cell lymphoma/leukemia‐2 protein
BPH: benign prostatic hyperplasia
BSO: buthionine sulfoximine
CAFs: cancer associated fibroblasts
ccRCC: clear cell renal cell carcinoma
CGH: comparative genomic hybridization
ChRCC: chromophobe renal cell carcinoma
COAD: colorectal adenocarcinoma
CRC: colorectal cancer
CRCLM: confined colorectal cancer liver metastases
CSS: cancer specific survival
CyTOF: mass spectrometry flow cytometry
DCC: distal cholangio carcinoma
DCs: dendritic cell
DFS: disease free survival
DSS: disease specific survival
ECM: extracellular matrix
EGFR: epidermal growth factor receptor
EMT: epithelial mesenchymal transition
EOC: epithelial carcinoma of the ovary
FDCs: follicular dendritic cell
GAR: GGT/albumin
GBM: glioblastoma multiforme
GC: gastric cancer
GCB‐DLBCL: diffuse large B‐cell lymphoma
GCs: germinal centers
GGT: gamma‐glutamyl transferase
GPR: GGT/Plt
GSEA: gene set enrichment analysis
GSH: glutathione
HBV: hepatitis B virus
HCC: hepatocellular carcinoma
HCV: hepatitis C virus
Her2: human epidermal growth factor receptor‐2
HIF‐1a: hypoxia‐inducible factor‐1a
HLF: hepatic leukemia factor
HNSCC: head and neck squamous cell carcinoma
ICC: intrahepatic cholangiocarcinoma
ICI: immune checkpoint inhibition
IHC: immunohistochemistry
IPMN: intraductal papillary mucinous neoplasia
LEPR: leptin receptor
LGG: low‐grade glioma
LGG: low‐grade glioma
LT: liver transplantation
LTC4: Leukotriene C4
LTD4: Leukotriene D4
LUAD: lung adenocarcinoma
MPM: malignant pleural mesothelioma
MRCC: metastatic renal cell carcinoma
mRCC: renal cell carcinoma
MRPS: metabolic related prognostic profile
NAC: N‐acetylcysteine
NET: new neuroendocrine tumor
NSCLC: non‐small cell lung cancer
OC: ovaries
ORFs: open reading frame
OS: overall survival
OSCC: oral squamous cell carcinoma
PA: pancreatic carcinoma
PAAD: pancreatic ductal adenocarcinoma
PAGE: polyacrylamide gel electrophoresis
PC: pancreatic
PCa: prostate cancer
PDACs: pancreatic ductal adenocarcinomas
PFS: progression free survival
PFS: progression free survival
PHC: primary hepatocellular carcinoma
PPS: survival after progression
pRCC: papillary renal cell carcinoma
PSMA: prostate specific membrane antigen
PTC: papillary thyroid carcinoma
RCC: renal cell carcinoma
RFS: recurrence free survival
ROS: reactive oxygen species
SASP: aging‐related secretory phenotypes
SMRP: serum mesothelin related protein
SNVs: single‐nucleotide variant
STAD: stomach adenocarcinoma
TACE: transcatheter Arterial Chemoembolization
TAMs: tumor associated macrophages
TAMs: tumor associated macrophages
TC: thyroid cancer
TC: thyroid cancer
TCF2: transcription factor 2
TGF‐β1: transforming growth factor‐β1
TMB: tumor mutation load
TNBC: triple‐negative breast cancer
TP53: tumor protein 53
Tregs: regulatory T‐cell

## 1 Introduction

In recent years, with the rapid development of bioinformatics, many novel biomarkers for the diagnosis and prognosis of many types of cancers have been discovered on the basis of large‐scale RNA sequencing (RNA‐seq) transcriptomic data and corresponding clinical follow‐up information. More efficient and accurate methods have facilitated the use of personalized medicine in clinical practice. Reprogramming of cellular metabolism is a hallmark of tumorigenesis, and changes in metabolism‐related genes lead to abnormalities in metabolism‐related pathways and metabolite production in tumor cells, which are associated with tumor transformation, growth, and progression. Specific metabolic activities have been developed for tumor imaging, providing prognostic biomarkers and identifying therapeutic targets. Therefore, exploring and exploiting specific metabolic alterations in cancer is important for clinical oncology and basic cancer pathophysiology. Accurate early diagnosis or prognostic assessment can be achieved on the basis of the association between GGT gene expression and corresponding phenotypic changes in tumors. The development of novel biomarkers in tumors will facilitate early diagnosis, guide therapeutic decisions, and provide potential therapeutic targets. Compared with the findings of Corti et al., our review is significantly improved in several aspects. The scope of research is broader, not only covering the role of GGT in antioxidative stress but also comprehensively analyzing the diversity of the GGT gene family from the perspective of genomics and transcriptomics, as well as its expression pattern, mechanism, and prognostic significance in multiple tumor types, such as hepatocellular carcinoma, colorectal carcinoma, gastric carcinoma, and breast carcinoma. Moreover, the complexity of the GGT gene family has been explored at multiple levels, and the aberrant expression and prognosis of GGT1, GGT2, GGT5, GGT6, GGT7, GGTLC1, GGTLC2, and GGTLC3 in multiple tumors, as well as the mechanism of transcriptional regulation, such as tumor‐specific expression of different promoter‐driven products, have been analyzed in detail. In terms of clinical application prospects, we have not only explored the basic biological functions and mechanisms of GGT but also focused on its clinical value. We analyzed the potential of GGT in tumor diagnosis, prognosis assessment, and therapeutic target development and pointed out the potential applications of GGT as a tumor marker and a GGT inhibitor, which has pointed out the direction of clinical research. In addition, the book incorporates the latest research progress and includes cutting‐edge content on the regulation of GGT in the tumor immune microenvironment, its relationship with metabolic reprogramming, and the application of serological tests, helping readers grasp the dynamics of GGT research in a comprehensive manner. In addition to traditional methods, bioinformatics analysis, big data mining, and other methods are used to systematically analyze the expression profiles of the GGT gene family and the correlation between mutation characteristics and clinicopathological parameters, which provides powerful support for in‐depth investigations of the role of GGT in tumors.

## 2 Genomic and Transcriptomic Regulation of GGTs

### 2.1 GGT Gene Family

The human GGT genes belong to a multigene family encoding enzymes involved in glutathione metabolism, amino acid transpeptidization for expression in different physiological states, and high physiological variability. The GGT family of genes plays an important role in the physiopathology of disease. Early pregenomic studies have shown that, in addition to the well‐characterized GGT1 gene, the human genome contains other related genes or sequences that have been given multiple different names, leading to inconsistency and confusion in the naming of the various members of the GGT gene family. Multiple alternatively spliced variants have been identified. Multiple related genes exist on chromosomes 20 and 22, and pseudogenes exist on chromosomes 2, 13, and 22. The human genome sequence contains 13 GGT homologs, and at least eight GGT genes or pseudogenes have been identified. The functional enzymes produced by GGT genes are all membrane‐bound sugar heterodimers that hydrolyze γ‐glutamyl, primarily glutathione (GSH) and glutathione couplings, under physiological conditions. The human GGT gene is located on chromosome 22, q11.1–q11.2, and there exists a q11–qter BCR gene phase region off the domain encoding an effective GGT. Three of these homologs, GGTLC1 (formerly known as GGTL6, GGTLA3, GGTLA4, dJ831C21.1, dJ831c21.2), GGTLC2 (formerly known as GGTL4), and GGTLC3 (formerly known as GGT), are three loci that may encode only the light chain portion of GGT and at least four other homologs of active genes with nucleotide or amino acid sequence similarity to GGT1: GGT2, GGT5 (formerly known as GGL, GGT‐REL, GGTLA1), GGT6 (formerly known as the rat GGT6 homolog), and GGT7 (formerly known as GGT4, GGTL3, GGTL5, D20S101). The human GGT2 gene expresses proteins that share 94% of the amino acid sequence encoded by GGT1, even though GGT2 encodes only inactive prepeptides. GGT2 is also aberrantly expressed in tumors, and the upregulation of GGT2 overcomes H_2_O_2_ induced apoptosis. GGT1 and GGT5 are expressed by different types of cells in tissues, and their different patterns of expression lead GGT1 and GGT5 to contact different extracellular fluids and thus different substrates. Studies have shown that GGT5 can contact substrates in blood and intercellular fluids, whereas GGT1 mainly contacts fluids in ducts and glands throughout the body [1]. Although GGT5 and GGT1 share 40% of the amino acid sequence, GGT5 is not as active as GGT1 in hydrolyzing GSH, GSSG, and LTC4. GGT1 and GGT5 have different substrate specificities; for example, the value of GSH is the same for GGT1 and GGT5 (11 μM), whereas the value of GSSG is different (9 μM for GGT1, GGT5 43 μM) [2]. Despite the lack of validated protein‐coding activity, GGT6 and GGT7 are highly expressed in tumors, pancreatic diseases, and other disorders, and these genes increase glutathione metabolism, γ‐glutamyl transfer, and leukotriene synthesis through their as yet uncharacterized enzymatic activities [3]. The GGT gene family has important tumor diagnostic value and is closely related to the development of iron death‐related tumors. The mechanism is as follows: (1) GGT decomposes extracellular GSH to release glutamate, cysteine, and glycine. Cysteine is the key precursor for GSH synthesis and can be taken up by the cell for the resynthesis of GSH. GGT also catalyzes the transfer of the γ‐glutamyl group of GSH to other molecules, regulates the metabolic balance of GSH/GSSG, enhances the antioxidant capacity of tumor cells, inhibits iron death, and promotes tumor cell survival and proliferation. GGT can also catalyze the transfer of the γ‐glutamyl group of GSH to other molecules, regulate the metabolic balance of GSH/GSSG, maintain intracellular GSH homeostasis, increase the antioxidant capacity of tumor cells, inhibit iron death, and promote the survival and proliferation of tumor cells. (2) GSH is an important substrate for glutathione peroxidase 4 (GPX4), which can reduce lipid peroxidation to the corresponding alcohols and inhibit lipid peroxidation and the initiation of iron death. GGT is the downstream target of GPX4. GGT is the downstream target of GPX4, and GPX4 affects the expression and activity of GGT family proteins by regulating the GSS/GSR complex [4]. (3) GSH can scavenge reactive oxygen species (ROS), preventing oxidative stress caused by the accumulation of ROS, and iron‐originating ROS can activate the NF‐κB pathway, increase its ability to bind to DNA, inhibit lipid peroxidation and the accumulation of ROS, and protect tumor cells from the effects of iron death [5]. (4) GGT can regulate the interaction between cystine and glutathione, activate the mTORC1 signaling pathway, inhibit the occurrence of the integrated stress response (ISR) and iron death, and promote the development of cancer [5]. For example, in clear cell renal cell carcinoma (ccRCC), GGT1 can maintain the level of intracellular GSH in ccRCC cells, protect against oxidative stress, and inhibit lipid peroxidation and iron death. It promotes ccRCC cell proliferation, migration, and tumor growth in vivo [6]; in glioblastoma, GGT1 resists cellular iron death caused by disruption of cellular redox homeostasis due to the absence of cystine and GSH [7, 8]; in triple‐negative breast cancer, increased expression of HLF increases GGT1 transcripts, increases the intracellular GSH content, increases the ratio of GSH to oxidized glutathione (GSSG), activates the iron death regulator GPX4 to effectively remove intracellular lipid reactive oxygen species (ROS), maintains the intracellular redox balance, inhibits iron death, and enhances the proliferation and metastatic ability of TNBC cells; moreover, tumor cells and tumor‐associated macrophages promote the continuous activation of hepatic leukemia factor through the interleukin‐6‐transforming growth factor‐β axis, and GGT1 promotes cisplatin tolerance in cells by increasing iron death resistance [9]. In pancreatic ductal adenocarcinoma (PAAD), TXNDC12 interacts with GGT7 through cysteine residues in its active site to activate GGT7, resulting in an increase in GSH, a decrease in GSSG, a decrease in reactive oxygen species (ROS) and malondialdehyde (MDA), and an increase in glutathione peroxidase 4 (GPX4) expression. Therefore, the occurrence of iron death can be inhibited [10]. When the TCGA database was utilized for bioinformatics analysis, we used a variety of statistical methods and algorithms to ensure the accuracy and reliability of the results. For example, Cox regression modeling was used to assess the relationship between gene expression and patient survival, and the false‐positive rate of multiple tests was controlled by FDR correction. Survival analyses were performed via the Kaplan–Meier method to plot survival curves, and survival differences between groups were compared via log rank tests. In addition, quantitative analysis of immune cell infiltration in tumor samples was performed via the CIBERSORT algorithm to assess the distribution of immune cells in different samples. The application of these methods and algorithms provides a solid methodological basis for studying the expression pattern and prognostic value of the GGT gene family in tumors (Table 1).

**TABLE 1:** GGT homologous genes.

Gene	Former name	Functional proteins	Abnormal expression	Primary expression organization	Located on A chromosome
GGT1	CD224, D22S672, D22S732, GGTD, GTG	Functional protein	Dysregulation in various tumors	Kidney, prostate, liver, duodenum, pancreatic, thyroid gland, etc.	22q11.23
GGT2		94% homologous to part of GGT1	Glioblastoma multiforme low expression	Brain, liver, lungs, testes, placenta, etc.	20q11.2
GGT5	GGL, GGT‐REL, GGTLA1	Functional protein is 40% homologous to GGT1, hydrolyses GSH < 1/46 of GGT1 activity, hydrolyses GSH, GSSG, LTC4	High expression of GGT5 favors the prognosis of GGT1 hepatocellular carcinoma	Stomach, kidneys, liver, intestines, fat, etc.	22q11.23
GGT6	Rat GGT6 homolog		Overexpressed in low‐grade gliomas	Brain, colon, kidneys, etc.	17p13.2
GGT7	GGT4, GGTL3, GGTL5, D20S101		Overexpression in low grade gliomas; high expression may lead to lower overall survival in hepatocellular carcinoma; GGT7 polymorphic loci rs6119534 and rs11546155 are associated with risk of pancreatic disease	Brain, liver, stomach, pancreas, mammary gland, etc.	20q11.22
GGTLC1	GGTL6, GGTLA3, GGTLA4, dJ831C21.1, dJ831c21.2	Encodes only the GGT portion of the light chain		Uterus, prostate, lungs, breast, etc.	20p11.21
GGTLC2	GGTL4	Encodes only the GGT portion of the light chain		Kidneys, testicles, etc.	22q11.22
GGTLC3	GGT	Encodes only the GGT portion of the light chain		Kidneys, lungs, etc.	22q11.21

### 2.2 Members of the GGT

GGT1: Also known as CD224, D22S672, D22S732, GGTD, GTG. The GGT1 gene is 2376 nucleotides in length, with a coding region of 1710 bases localized to the long arm of human chromosome 22 at 11.23 (22q11.23), and encodes 569 amino acids. GGT1 activity is high, and GGT1 is the key gene encoding the membrane‐bound enzyme GGT1 protein, which is most widely expressed in human tissues. The GGT1 gene is commonly expressed in the liver, kidney, duodenum, and 11 other tissues involved in uptake and secretion. Studies have shown that GGT1 is involved in the development of clear cell renal cell carcinoma by promoting tumor cell migration and that the inhibition of GGT1 significantly attenuates migration, suggesting its therapeutic potential [6, 11]. In addition, serum exosomal GGT1 can be used as a marker for the clinical features of advanced renal carcinoma [12], and a similar pattern was observed in prostate cancer, where serum exosomal GGT1 activity was significantly greater in malignant patients than in patients with benign hyperplasia [13]. GGT1 also has differential roles in other cancers (e.g., its regulatory association with TP53/BCL2 in breast cancer [14] is correlated with immune infiltration and methylation in hepatocellular carcinoma [15]). GGT1 deficiency is a rare autosomal recessive disorder that can lead to disturbances in glutathione homeostasis, weight loss, reproductive defects, mental retardation, and cataracts (in the absence of GGT, the absence of GSH in the body leads to a depletion of cysteine, which is not compensated for by hepatic synthesis; the lack of cysteine limits glutathione synthesis, and the concentration of glutathione in the eye is only 39% of normal, leading to free radical accumulation and the induction of cataract formation). Liebermann and coworkers reported the phenotype of GGT1 knockout mice, and GGT1 deficiency has been shown to attenuate oxidative stress and increase susceptibility to oxidative stress and lung injury in GGT1 knockout mice compared with wild‐type mice. Compared with wild‐type mice, GGT1‐deficient mice presented significantly higher plasma (175 μM) and urine (15.4 μM) GSH concentrations, as well as growth retardation and cysteine deficiency (plasma and urinary GSH concentrations of 27.6 M and 6.2 μM, respectively), suggesting that the involvement of GGT1 is critical for the hydrolysis of extracellular GSH and the sustained provision of cysteine to cells. This finding also confirms that the GGT1 knockout mouse phenotype can be rescued by supplementing dietary water with N‐acetylcysteine (NAC) [16]. Typically, patients with GGT1 deficiency have significantly elevated blood glutathione levels above the renal threshold for glutathione recovery, which can lead to glutathionuria, in which these patients secrete glutathione in the urine but fail to metabolize LTC4. Only a few relevant cases have been reported to date, and they have milder phenotypes and different symptoms than mice do because of the presence of other functional GGT genes.

GGT2: The GGT2 gene is located on human chromosome 20 (20q11.2), which encodes a γ‐glutamyltransferase (AKA γ‐glutamyltransferase enzyme, EC 2.3.2.2) and is highly expressed in tissues, mainly in the testis, brain, and placenta. GGT2 is aberrantly expressed in certain tumors (e.g., testicular cancers and gliomas) and may be associated with tumor progression and drug resistance [15].

GGT5: This gene is also known as GGL, GGT‐REL, and GGT like activity 1 (GGTLA1), and the GGT5 gene is located on human chromosome 22 (22q11.23). It cleaves the γ‐glutamyl portion of glutathione and plays a key role in redox regulation, immune function, and drug metabolism. The protein encoded by this gene is synthesized as a single catalytically active polypeptide, which is posttranscriptionally processed to form a small and large subunit within which the catalytic activity is contained. GGT5 is a γ‐glutamyl leukotriene that can convert leukotriene C4 (LTC4, a glutathione S‐suffix) to leukotriene D4, which plays a key role in the pathogenesis of asthma and in the immune system. Because GGT5 cannot cleave γ‐glutamyl‐p‐nitroanilide, its activity cannot be detected by conventional methods, but it has a unique substrate specificity compared with that of γ‐glutamyltransferase, and alternative splicing results in multiple transcript variants encoding different isoforms. Because GGT5 cannot cleave GSH, GGT5‐deficient patients exhibit a normal phenotype. GGT5 is widely expressed in adipose, kidney, liver, and 23 other tissues. Many tissue‐localized macrophages, such as Kupffer cells in the liver and alveolar macrophages, express the GGT5 protein. Wei et al. reported that GGT5 is highly expressed in cancer‐associated fibroblasts in lung cancer, which predicts decreased survival in lung cancer patients [17]. In addition, GGT5 is considered a key metabolism‐related gene in gastric cancer, colon cancer, ovarian cancer, and B‐cell lymphoma and is associated with tumor progression and poor prognosis. GGT5 contributes to tumor development by participating in a variety of signaling pathways, acting on immune cell infiltration in the tumor microenvironment, and regulating the metabolism of tumor cells. However, GGT5 acts as a tumor suppressor in hepatocellular carcinoma, breast cancer, and clear cell renal cell carcinoma. This discrepancy may be related to a variety of factors, such as differences in the tumor microenvironment, the complexity of gene expression regulatory mechanisms, and interactions with other signaling pathways, and further in‐depth follow‐up studies are needed to clarify its specific mechanisms and exact roles in different cancers.

GGT6: The GGT6 gene is located on human chromosome 17 (17p13.2), and GGT6 is commonly expressed in the colon, kidney, and 12 other tissues. There are few studies on GGT6 in the field of cancer. Two recent studies emphasized the prognostic role of GGT6 mRNA in head and neck squamous cell carcinoma and papillary renal cell carcinoma [15, 18, 19].

GGT7: GGT7 is also known as GGT4, GGTL3, GGTL5, and D20S101, and the GGT7 gene is located on human chromosome 22 (22q11.22). Compared with GGT1 and GGT5, which have known functions, GGT7 is a new member of the GGT family with 47% and 52% amino acid sequence similarity, respectively. It is ubiquitously expressed in the brain, thyroid, liver, and 24 other tissues; encodes a member of a family of enzymes involved in glutathione metabolism and amino acid transpeptidation; and is highly expressed in the brain, with the lowest expression in gliomas compared with normal brain tissues. A study related to GGT7 and glioblastomas suggested that GGT7 may play a key role in promoting gliosis by regulating reactive oxygen species (ROS) levels during the growth of glioblastoma by regulating reactive oxygen species (ROS) levels, which play a key role in the growth of glioblastoma [20]. Changes in γ‐glutamyltransferase activity may signal a pretumorigenic or toxic status in the liver or kidney. Tian S first explored the oncogenic role of GGT7 in HCC and confirmed that the expression of GGT7 mRNA in hepatocellular carcinoma tissues was greater than that in normal liver samples and that high expression of GGT7 mRNA was associated with poorer overall survival (OS) and poorer disease‐free survival (DFS) and that GGT7 may be a promising biomarker for predicting the survival of patients with HCC [15]. GGT7 may also be involved in the interaction of the lung cancer progression related plasma membrane associated protein CT120.

GGTLC1: GGTLC1 is also known as GGTL6, GGTLA3, GGTLA4, dJ831C21.1, and dJ831c21.2. The GGTLC1 gene is located on human chromosome 20 (20q11.21), and the product of the gene is homologous to only the light chain region and lacks the transmembrane region. Multiple alternatively spliced variants encoding the same protein have been identified. GGTLC1 plays important roles in regulating the cellular redox state, the cell cycle, and chemotherapeutic drug efflux. Bias expression in the lung, testis, and 6 other tissues.

GGTLC2: GGTLC2 is also known as GGTL4, and the GGTLC2 gene is located on human chromosome 22 (22q11.22), which encodes a protein related to the enzyme that cleaves the γ‐glutamyl peptide bond in glutathione and other peptides. Unlike similar proteins, the encoded protein contains only light chain portions and may not be catalytically active. Selective cleavage results in multiple transcriptional variants. The gene has multiple related family members and related pseudogenes located in the same region of chromosome 22. Biased expression is observed in the kidney, testis, and 10 other tissues.

GGTLC3: GGTLC3 is also known as GGT, and the GGTLC3 gene is located on human chromosome 22 (22q11.21). This gene has only light chain GGTs; GGTLC3 contains a region corresponding to the light chain of GGT1, but it lacks the membrane‐anchored heavy chain region (Table 2).

**TABLE 2:** Abnormal GGT expression in tumors and associated mechanisms.

Gene	Cancer type	Machanisms
GGT1	Clear cell renal cell carcinoma (ccRCC)	Clear cell renal cell carcinoma (ccRCC) is the most common subtype of renal cancer, Zhang Bing et al. found that GGT1 was different in plasma, cancerous tissues and normal renal tissues of ccRCC patients by microarray analysis and bioinformatics analysis, and that GGT1 was highest in the cell lines from which ccRCC tumors originated. gGT1 is a potential marker for ccRCC [11]. Bansal, et al. Human knockdown of GGT1 inhibited the proliferation of ccRCC cells, and in vivo experiments showed that GGT1 promoted the proliferation of ccRCC, and GGT1 deficient ccRCC cells formed tumors that were significantly smaller in volume and weight than those of the control group, with fewer Ki67 positive cells in the tumor tissue. GGT1 promoted ccRCC cell migration as observed by scratch and transwell assays. GGT1 is expected to be a therapeutic target for the treatment of ccRCC, overcoming the chemoresistance of ccRCC and inhibiting the proliferation and migration of tumor cells, thus inhibiting tumor progression and metastasis [6].
	Chromophobe renal cell carcinoma (ChRCC)	Smoky renal cell carcinoma (ChRCC) accounts for 5% of all sporadic renal cancers, and the expression level of GGT1 mRNA in ChRCC is 100‐fold lower than that in normal kidneys and 70‐fold lower than that in the protein level, suggesting that reduced GGT1 expression may be one of the metabolic features specific to ChRCC and may serve as a potential marker to differentiate ChRCC from other types of renal cancers. Low GGT1 expression leads to increased intracellular GSH levels, which enhances cellular resistance to oxidative stress and promotes cell growth. The therapeutic use of H_2_O_2_ and buthionine sulfoximine (BSO), an inhibitor of GSH synthesis, significantly inhibited ATP production in GGT1 overexpressing cells, providing a potential pathway for this rare tumor, which is uniquely without a specific targeted therapy at present [21, 22].
	Renal cell carcinoma (RCC)	It was found that the expression of GGT1 was upregulated in renal cancer tissues, and high levels of GGT1 affected intracellular redox homeostasis by regulating GSH cycling, which in turn affected HIF‐1α signaling, leading to increased expression of genes such as VEGFA, reprogramming of RCC metabolism, angiogenesis, tumor progression, and resistance to chemotherapy [23]. The upregulation of GGT1 may be related to the antioxidant capacity of renal cancer cells and the metabolic status, which in turn affects tumor progression, and GGT1, as one of the differentially expressed genes, may be a potential target for renal cancer research [12]. Horie et al. applied immunohistochemistry to analyze surgically resected RCC specimens, and found that the expression of GGT1 was increased in the RCC membranes with microvessel invasion, and that the preoperative serum exosomal GGT1 activity predicted the pathological diagnosis of microvessel in the surgically resected specimen invasion in surgically resected specimens. Serum exosomal GGT1 activity is associated with advanced renal cell carcinoma invasion and metastasis, and can be a clinically useful marker for clinicopathologic features, which can be used in combination with traditional diagnostic methods to improve the diagnosis and potential therapeutic targets in patients with renal cell carcinoma (RCC) [13].
	Prostate cancer (PCa)	Kawakami et al. upregulated GGT1 expression in exosomes isolated from C4‐2 and C4‐2B cells by differential centrifugation and immunocapture with anti‐CD9 or prostate‐specific membrane antigen (PSMA) antibodies, and analyzed the exosomes by histochemical staining in PC tissues and benign prostatic hyperplasia (BPH) tissues, respectively, which showed that GGT1 was overexpressed in PC plasma membrane and that the GGT1 activity in serum exosomes of PC patients was significantly higher than that of prostate hyperplasia patients as detected by ELISA and fluorescent probe, and serum exosomal GGT1 activity has the potential to become a new diagnostic biomarker for PC [24].
	Hepatocellular carcinoma (HCC)	Tian S did not find differential expression of GGT1 mRNA in hepatocellular carcinoma specimens and pathologically normal tissues, but its low expression may predict relatively good overall survival (OS) in patients, and this correlation disappeared in disease‐free survival (DFS). The expression of GGT1 was negatively correlated with the level of DNA methylation and correlated with the infiltration of multiple immune cells. These findings suggest that GGT1 may have a role in the progression of HCC, and therefore, serum GGT protein levels may serve as a potential biomarker for assessing the survival outcome of patients with hepatocellular carcinoma [15].
	Gastric cancer (GC)	GGT1 is associated with the prognosis of gastric cancer, and studies have shown that high expression of GGT1 correlates with lymph node metastasis, histologic type, and expression of human epidermal growth factor receptor 2 (Her2) in gastric cancer, and is higher in papillary and tubular adenocarcinomas. Kaplan Meier et al. suggested that high expression of GGT1 significantly affects the overall survival of patients with gastric cancer through a survival analysis, and Cox risk modeling analysis showed that GGT1 can be used as an independent predictor of poor prognosis in patients with gastric cancer, and it can better predict the poor prognosis of patients with progressive, poorly differentiated, undifferentiated, mucinous adenocarcinoma+Indicator cell carcinoma, and HER2‐positive gastric cancer. GGT1 is expected to serve as a new marker in the diagnosis and prognostic evaluation of gastric cancer, and with the in‐depth study of the function and mechanism of action of GGT1, it may provide a new target in the treatment of gastric cancer [25].
	Pancreatic (PC)	5%–10% of pancreatic cancers are Lewis antigen‐negative and have sparse CA19‐9 secretion and defective fucosylation, an aggressive subgroup with specific clinical and molecular features. Potential proteoglycans have been identified in Lewis‐positive pancreatic cancers, and GGT1, along with EGFR, HSPG2, ADAM17, GPC1, ITGA2, CD40, and IL6ST, have been identified as potential interacting proteins, suggesting that GGT1 may play a role in signaling and cellular functions in pancreatic cancer cells [26]. Agawa, S. et al. found that CPA1 and GGT1 genotypes may be potential tools for diagnosing pancreatic intraductal papillary mucinous neoplasia (IPMN) and predicting its malignant features [27]. GGT1 gene variants can affect pancreatic cancer risk as well as prognosis and treatment outcome [28].
	Colorectal cancer (CRC)	TNM stage is a potential prognostic factor for tumor patients, and the higher the stage, the worse the prognosis. GSK3B, GGT1, and EIF2B5 are three new driver genes in stage IV progressive colorectal cancer. High expression of GGT1 is associated with malignant phenotype and poor prognosis of tumors, and colorectal cancer stage IV can be predicted and colorectal cancer prognosis can be evaluated based on the expression of GGT1. GGT1 provides a new metabolic pathway for CRC cancer cells to adapt to hypoxic environment and maintain survival under chronic hypoxic conditions through γ‐glutamyl cycle. By inhibiting the activity of GGT1, the uptake and metabolism of amino acids by cancer cells are reduced, thus inhibiting their growth and proliferation, and targeting GGT1 may become a potential strategy for the treatment of colorectal cancer under chronic hypoxic conditions. GGT1 is a potential diagnostic biomarker of colorectal cancer as well as a novel stage‐specific pharmacotherapeutic target for rational intervention [29, 30].
	Thyroid cancer (TC)	In thyroid cancer tissues, the percentage of GGT1 mutations was significantly higher than that of suspected benign nodules, and GGT1 mutations may play an important role in the development of thyroid cancer, and it is expected to be one of the potential molecular markers to identify benign and malignant nodules of the thyroid gland. The positive correlation between GGT1 mutations and lymph node metastasis of thyroid cancer may play an important role in the process of progression and metastasis of thyroid cancer, and it may be a potential clinical value for evaluating the prognosis and treatment plan of patients with thyroid cancer. It has potential clinical value in evaluating the prognosis of patients with thyroid cancer, disease progression, and formulating therapeutic plans [31, 32]. Zhang et al. Combining genetic alterations with clinical and radiological features significantly improves the predictive validity of radiological features for lymph node metastasis, and targeted sequencing of comutations in papillary thyroid carcinoma (PTC) driver genes (e.g., GGT1, BRAF, TERT) has high prognostic value. GGT1 is expected to serve as a diagnostic marker and a therapeutic target for PTC, and its detection can help identify aggressive PTC subtypes and improve diagnostic accuracy [33].
	Glioblastoma (GBM)	Genome sequencing of diffuse astrocytic gliomas by Tunthanathip et al. revealed the presence of mutations in the GGT1 gene. GGT1 resists cellular iron death caused by disruption of cellular redox homeostasis due to cystine and GSH deficiency, and upregulation of GGT1 increases the level of intracellular GSH, which protects the tumor cells from evasion of oxidative stress and promotes the growth of tumor cells [7]. Under hypoxic conditions, GGT1 activates Rho GTPase by localizing it to the cell membrane through isoprenylation modification, which in turn induces the expression and transcriptional activity of the oxygen‐inducible factor 1a (HIF‐1a), thereby activating multiple genes and promoting the proliferation, migration and invasion of glioblastoma cells. Studies have shown that GGT1‐specific inhibitors such as GGTI‐298 can reduce the level of HIF‐1a protein and inhibit the migration and invasion ability of tumor cells, thereby slowing down tumor progression [34]. GGT1 may serve as a glioblastoma biomarker for predicting glioblastoma sensitivity to iron death‐inducing agents, as well as for assessing patient prognosis and response to therapy [8].
	Non‐small cell lung cancer (NSCLC)	GGT1 is highly expressed in the A549 lung adenocarcinoma cell line and its expression increases after IL‐1β stimulation, which rapidly converts LTC4 to LTD4 [35], and GGT1 shows an important role in the development of resistance to erlotinib in non‐small cell lung cancer (NSCLC) cells [36].
	Triple‐negative breast cancer (TNBC)	Choi, J found that the positive expression rate of GGT1 in TNBC was 18.9%, which is not universally highly expressed in TNBC but has certain expression specificity. GGT1, as one of the molecular phenotypic markers of TNBC, can help to further subdivided TNBC into different subtypes, which can potentially provide a basis for individualized treatment and prognostic assessment. The expression of GGT1 in TNBC may constitute a comprehensive molecular marker spectrum together with other tumor markers (e.g., EGFR, CK5/6), providing clues for a deeper understanding of the molecular mechanisms and clinical features of TNBC. The expression profile of GGT1 in TNBC may, together with other tumor markers (e.g., EGFR, CK5/6, etc.), constitute a comprehensive molecular marker profile, providing important clues for an in‐depth understanding of the molecular mechanisms and clinical features of TNBC [37]. Transforming growth factor β1 (TGF‐β1) secreted by tumor associated macrophages (TAMs) regulates hepatic leukemia factor (HLF), and IL‐6 produced by triple‐negative breast cancer (TNBC) cells activates the JAK2/STAT3 axis inducing TGF‐β1 to constitute a feed‐forward loop, and the interactive dialog between TNBC cells and TAMs promotes through the IL‐6‐TGF‐β1 axis the Sustained activation of HLF in tumor cells. transactivation of GGT1 by HLF promotes iron death resistance in TNBC cells and drives proliferation, metastasis, and cisplatin resistance in TNBC cells of triple‐negative breast cancer, and the combination of HLF and GGT1 improves the prognostic accuracy of TNBC patients, providing a potential target for the treatment of TNBC [9]. Ding et al. found that GGT1 promotes tumor stem cell migration and invasion in breast cancer by activating the NF‐κB signaling pathway to activate downstream genes (the p65 subunit of NF‐κB binds to the promoter region of GGT1 and promotes its transcription to form an NF‐κB/GGT1 positive feedback loop), and correlates with chemo‐resistance and poor prognosis. High GGT1 expression is significantly correlated with breast cancer The high expression of GGT1 is significantly associated with tumor stage, lymph node metastasis and shortened overall survival in patients, and can be used as a poor marker for breast cancer prognosis [38]. Sun Lu et al. found that pretreatment serum GGT levels and polymorphisms in its encoding gene GGT1 (GGT1 SNP) can be used as potential biomarkers for predicting the efficacy, prognosis, and side effects of neoadjuvant chemotherapy for breast cancer [39].
	Parietal plasma‐secreting breast cancer	GGT1 is significantly expressed in Cowden's disease breast cancer. Studies have shown that in the early stage of the tumor, PTEN deficiency activates the ERBB2‐PI3K‐AKT pathway, which promotes the formation of breast tumors with apocrine gland features. The expression of GGT1 may be associated with the ERBB2‐PIK3CA‐PTEN‐AKT signaling pathway. This signaling pathway plays an important role in the development of breast cancer, and the expression of GGT1 may reflect the aberrant activation of this pathway. GGT1 can be used as a potential marker for identifying breast cancers with acinar features, and may provide a new target for future diagnosis and treatment [14].
	Ovarian cancer (OC)	Studies have shown that the expression of GGT1 is significantly higher in ovarian cancer tissues than in normal ovarian tissues, especially in ovarian epithelial carcinoma (EOC), and correlates with the stage and grade of the tumor. The high expression of GGT1 is associated with tumor proliferation, invasion, metastasis, and drug resistance. Although CA125 is currently the most commonly used biomarker for ovarian cancer, it has low sensitivity in early‐stage ovarian cancer and may be elevated in some benign diseases. GGT1, as a new biomarker, may be potentially valuable in complementing CA125, especially in the detection of early‐stage ovarian cancer. The expression level of GGT1 can be used as a potential biomarker for ovarian cancer marker for early detection and prognostic evaluation of ovarian cancer [40].
GGT2	Glioblastoma multiforme (GBM)	GGT2 showed significant prognostic value in both low‐grade glioma (LGG) and glioblastoma (GBM). The downregulation of GGT2 expression in LGG and GBM suggests that GGT2 may play an important role in the genesis and progression of gliomas. In LGG, the downregulation of GGT2 expression may be correlated with the early stage of tumor progression, whereas in GBM, the downregulation of its expression may be correlated with the late progression of the tumor and the increase in the degree of malignancy. GGT2 is expected to be an early detection of gliomas and prognostic assessment of potential biomarkers. In addition, the interaction of GGT2 with other genes, such as KRT75, may also have an impact on tumor progression in gliomas. The combined effects of these genes may provide new perspectives for the diagnosis and treatment of gliomas [41].
	Prostate Cancer (PCa)	K‐RAS transformation increases the level of GGT2 expression by activating the K‐RAS/ERK pathway.267 GGT2 expression in B1/K‐RAS cells contributes to resistance to H_2_O_2_ induced cell death. These results may facilitate the development of new anticancer therapies for K‐RAS oncogene‐induced cancers such as prostate cancer [42].
GGT5	Gastric cancer (GC)	Compared with normal gastric mucosa, GGT5 expression was upregulated in gastric cancer tissues, and multivariate analysis showed that high expression of antioxidant‐related gene GGT5 was significantly correlated with T stage, histological type, and histological grading, and GGT5 is expected to be used as a noninvasive biomarker for the early diagnosis of gastric cancer, especially in distinguishing early gastric cancer from normal gastric tissues with high sensitivity and specificity. In addition, GGT5 is an independent risk factor for poor prognosis of gastric cancer patients by multivariate analysis and nomogram modeling of overall survival in gastric cancer, and gastric cancer patients with high expression of GGT5 have poorer prognosis than those with low expression of GGT5 in terms of overall survival (OS), progression‐free survival (FPS), postprogression survival (PPS) and disease‐specific survival (DSS) [43, 44, 45, 46]. Chen et al. Gene set enrichment analysis (GSEA) using TCGA data showed that GGT5 expression was significantly associated with epithelial mesenchymal transition (EMT)‐related genes, and Western blot analysis showed that silencing of GGT5 increased the protein expression level of the epithelial marker, E‐cadherin. The metabolic pathways involved in GGT5 include arachidonic acid metabolism, The metabolic pathways involved in GGT5 include arachidonic acid metabolism, cytochrome P450 metabolism of xenobiotics, which may provide the material basis and energy support for gastric cancer development. GGT5 was positively coexpressed with immune‐related genes and immune checkpoint genes, and functional analysis showed that the differentially expressed genes related to GGT5 were mainly involved in the biological processes of immune and inflammatory responses, and were involved in the process of extracellular matrix and regulation of immune cells (especially tumor‐associated macrophages) in the tumor microenvironment. (especially tumor‐associated macrophages (TAMs), dendritic cells (DCs), CD4+ T cells and CD8+ T cells) infiltration in the tumor microenvironment, and contribute to the development and prognosis of gastric cancer by affecting the immune system.GGT5 may be involved in the regulation of vascular development, angiogenesis, cell motility and migration, and positively correlates with the PI3K/AKT pathway, and the in vitro experiments showed that GGT5 directly targets the PI3K/AKT pathway by Direct targeting of the PI3K/AKT‐MAPK‐MMPs pathway mediates GC cell proliferation, migration and invasion [47]. Functional enrichment analysis showed that GGT5 and its coexpressed genes are mainly involved in extracellular matrix (ECM)‐related processes, such as the organization of the extracellular matrix and cellular response to growth factor stimulation. The expression of GGT5 may be correlated with the tumor mutational load (TMB), which refers to the number of mutations in tumors, and a high TMB is usually correlated with the tumor's heterogeneity, immunogenicity, etc. It offers a potential basis for personalized treatment, GGT5 is expected to be a potential target for immunotherapy of gastric cancer. GGT5, as a promising prognostic biomarker of gastric cancer, provides a potential target for early diagnosis, prognostic assessment and immunotherapy of gastric cancer) [48, 49].
	Stomach adenocarcinoma (STAD)	Patients with advanced gastric adenocarcinoma (STAD) usually have high mortality and poor prognosis. Several databases and in vitro experiments have shown that the expression level of GGT5 in gastric cancer tissues is significantly higher than that in normal gastric tissues. Fundamental metabolic changes promote the growth and invasiveness of gastric adenocarcinoma, and GGT5 expression was significantly associated with later T stage (T2, T3, and T4) and higher tumor grade, pathological stage (II, III, and IV) and increased immune cell infiltration, and overall survival (OS) and disease‐free survival (DFS) were poorer in high‐expression gastric adenocarcinomas, and GGT5 was an independent poor prognostic marker. Gene set enrichment analysis (GSEA) showed that GGT5 overexpression may be involved in the JAK–STAT signaling pathway, MAPK signaling pathway, leukocyte transendothelial migration, and melanoma‐associated pathway, which are closely related to tumor proliferation, invasion and metastasis. Highly expressed GGT5 was closely associated with immune cell infiltration in the tumor microenvironment, and the infiltration levels of M2‐type macrophages, regulatory T cells (Tregs) and monocytes were higher in the high‐expression GGT5 group, whereas M2‐type macrophages and Tregs were correlated with the gastric adenocarcinoma immune‐suppressive microenvironment, which promotes tumor progression; and the levels of plasma cells and M1‐type macrophages were higher in the low‐expression GGT5 group, while the levels of M1 macrophages were associated with a favorable prognosis. The high expression levels of immune checkpoint proteins PD‐1 and CTLA‐4 in the high‐expression GGT5 group suggested that STAD patients with high GGT5 expression may have a better response to immunotherapy. Prognostic univariate and multivariate analyses showed that GGT5 is a potential prognostic factor and promising marker for immunotherapy response in gastric adenocarcinoma (STAD) [50, 51, 52].
	Hepatocellular carcinoma (HCC)	GGT5 expression may be affected by single nucleotide variants (SNVs) while fulfilling SNV score‐related gene criteria. HTRA3, GGT5, RCAN2, and LGA predicted HCC outcome, and these key differentially expressed gene constructs provided new insights into individual treatment [53]. Tian S found that GGT5 was differentially expressed in HCC by UALCAN analysis. Correlation analysis GGT5 was positively correlated with GGT1 and GGT6, and high expression of GGT5 mRNA was associated with longer disease‐free survival (DFS) in HCC patients, but not significantly correlated with overall survival (OS). DNA methylation of GGT5 was significantly higher in normal tissues than in corresponding HCC samples, and was negatively correlated with mRNA expression. GGT5 was significantly correlated with tumor purity and positively correlated with the infiltration levels of CD8+ T cells, CD4+ T cells, macrophages, neutrophils, and dendritic cells, promoting tumor proliferation and progression, with potential value for the prognosis of HCC [15]. Lou Yu et al. verified that GGT5 is an important regulator in the immune microenvironment of hepatocellular carcinoma by mass spectrometry flow cytometry (CyTOF) on tumor tissues from hepatocellular carcinoma patients and in vivo experiments in mouse models, that it can affect the infiltration and function of immune cells within the tumors, especially the T cells, and that overexpression of GGT5 and the metabolite leukotriene D4 can effectively prevent the depletion of the tumor‐infiltrating T lymphocytes and thus enhance the antitumour immune response. depletion of infiltrating T lymphocytes, thereby enhancing antitumour immune responses. Tumor tissues were analyzed using transcriptome sequencing to identify gene expression patterns associated with immune cell infiltration. GGT5 expression correlates with clinicopathological factors such as tumor size, degree of differentiation, and patient prognosis. GGT5 may serve as a potential biomarker for predicting the prognosis of hepatocellular carcinoma patients and their response to immunotherapy. Therapeutic strategies targeting GGT5 may help to enhance the efficacy of immunotherapy and improve the prognosis of hepatocellular carcinoma patients [54].
	Adenocarcinoma of the lungs (LUAD)	It was found that GGT5 was highly expressed in cancer‐associated fibroblasts (CAFs) in the lung adenocarcinoma (LUAD) microenvironment but not in tumor cells, and promoted tumor cell proliferation and drug resistance by regulating the tumor microenvironment. In addition, the overall survival (OS) and progression‐free survival (PFS) of LUAD patients with high expression of GGT5 were significantly shortened, which was closely related to the poor prognosis of patients. Cell growth, lesion formation, and sphere formation analyses showed that proliferation of adenocarcinoma‐associated fibroblasts (CAFs) was attenuated in GGT5 knockdown adenocarcinomas, and that high expression of GGT5 contributes to cancer cell survival and enhances tumor cell tolerance to chemotherapeutic agents (e.g., paclitaxel and cisplatin) by increasing intracellular glutathione and decreasing intracellular reactive oxygen free radicals (ROS), suggesting that GGT5 could be a promising therapeutic target [17].
	Non‐small cell lung cancer (NSCLC)	Studies have shown that in EGFR‐TKI‐resistant non‐small cell lung cancer (NSCLC) cells, the copy number of the GGT5 gene is significantly increased and the activity of glutamyltransferase is enhanced, and this metabolic remodeling makes cancer cells dependent on glutamine metabolism, which helps cancer cells to maintain their antioxidant capacity and detoxification function, and provides energy and biosynthesis precursors for the proliferation and survival of the cells. GGT5 gene Synergistic testing with other related genes (e.g., MYC, GSTT2, and GGT1) has been utilized as a biomarker for predicting patient response to EGFR‐TKIs as well as a novel target for therapy [35].
	Colon	In colon cancer, the expression level of GGT5 novel metabolic genes may be associated with metabolic reprogramming of tumors, shaping the tumor immune microenvironment, affecting nutrient uptake and utilization by tumor cells, and predicting overall survival of colon cancer patients and serving as a biomarker for colon cancer [55]. Wang et al. found that GGT5 was predominantly expressed in stromal cells with increased abundance in endothelial cells and mononuclear lineage cells by analysis of single‐cell RNA sequencing data, which may affect immune cell infiltration and function by influencing stromal cell‐tumor cell interactions. Response to immune checkpoint inhibition (ICI) therapy revealed that the expression level of GGT5 might serve as a potential marker for predicting the sensitivity of patients with rectal adenocarcinoma (COAD) to conventional chemotherapeutic agents. Novel Lipid Metabolism‐Based Signatures Generated from PIK3CG, GGT5, and PTGIS Predict Prognosis and Immunotherapy Response in COAD [56].
	Breast cancer (BC)	EMP3 and GGT5 are LARS regulated tumor suppressors in breast cancer, LARS a specific tRNA synthetase, LARS downregulation promotes breast tumor formation, GGT5 expression is subject to LARS‐mediated translational regulation, and in breast cancer, GGT5 overcomes chemo‐resistance non‐cell‐autonomously, and Passarelli, M. et al. Knockdown of GGT5 can enhance organoid growth, which further emphasizes its role as a tumor suppressor [57].
	Ovaries (OC)	By using human proteome microarray technology, GGT5 was screened as a potential ovarian cancer‐associated antigen by Yaru Duan et al. Indirect ELISA was applied to further verify that the expression level of GGT5 was higher in the ovarian cancer group than in the normal control group, and that GGT5 may play an important role in the development of ovarian cancer, and the change in its expression level may be a potential biomarker for the diagnosis of ovarian cancer. GGT5 may play an important role in the development of ovarian cancer. The logistic regression model based on three anti‐TAAs autoantibodies (VCL, GGT5 and TRIM21) was able to distinguish ovarian cancer from normal control or benign ovarian diseases. The detection of anti‐GGT5 autoantibodies has potential clinical applications in the early screening, diagnosis and monitoring of ovarian cancer, especially the combination of three anti‐TAAs autoantibodies (VCL, GGT5 and TRIM21) and CA125 can improve the diagnostic accuracy and sensitivity of ovarian cancer [58].
	Clear cell renal cell carcinoma (ccRCC)	Clear cell renal cell carcinoma (ccRCC) is a malignant tumor of the urinary system. Renal vein or vena cava thrombosis can be found in a subset of ccRCC patients and lead to deterioration of prognosis. Shi et al. verified that the expression of GGT5 was progressively decreased in normal tissues, tumor tissues, and renal vein or vena cava thrombosed tissues by Western blot and immunohistochemistry (IHC). Based on the different expression patterns between normal tumor thrombus triad, GGT5 plays a consistent inhibitory role in tumorigenesis and thrombus invasion. GGT5 plays a consistent inhibitory role in tumorigenesis and thrombus invasion, and downregulation of GGT5 expression may be associated with increased proliferation and invasion of tumor cells. GGT5 upregulation may contribute to the reduction of thrombus growth and progression, and decreased GGT5 expression is associated with poor prognosis of patients [59, 60].
	B‐cell lymphoma (BCL)	High expression of GGT5 in follicular dendritic cells (FDCs) contributes to the formation of a GGG concentration gradient, ABCC1 transports GGG to the extracellular compartment, and GGT5 breaks down GGG to inactivate it, which in turn restricts P2RY8‐mediated B cells to follicular center regions (GCs) and affects their migration inhibition and growth regulation functions, GGT5 may play a certain role. Since P2RY8 has been implicated in a variety of B‐cell lymphomas (e.g., diffuse large B‐cell lymphoid (GCB‐DLBCL), Burkitt's lymphoma [BL]) are frequently mutated, and thus the role of GGT5 in regulating P2RY8 receptor function and the microenvironment of B cells in the germinal centers, the combination of drugs targeting GGT5 and ABCC1 may regulate the distribution and function of GGGs more efficiently and provide a new strategy for the treatment of B‐cell lymphomas [61, 62].
GGT6	Head and neck squamous cell carcinoma (HNSCC)	The expression levels of GGT6 differed in normal oral epithelial cells and head and neck squamous cell carcinoma (HNSCC) cells, suggesting that it may be involved in tumorigenesis and progression. The expression level of GGT6 in HNSCC may affect the function of immune cells or the degree of infiltration, which may influence the immune response and prognosis of the tumor, and GGT6 has been considered as a potential prognostic marker. Therefore, GGT6 could be an important gene for studying the metabolic and immune characteristics of HNSCC and provide a potential target for future therapeutic strategies [18]. It was shown that the expression level of GGT6 was significantly upregulated in HPV+ oropharyngeal cancer patients, and its expression level was significantly correlated with patients' overall survival (OS) and disease‐free survival (DFS). Due to the differential expression of GGT6 in HPV+ oropharyngeal cancer and its correlation with prognosis, it may be a potential prognostic marker [63]. In oral squamous cell carcinoma (OSCC), the expression level of GGT5 is usually upregulated, and GGT5 plays a key role in the degradation and remodeling of extracellular matrix, which contributes to the proliferation, invasion, and metastasis of OSCC tumor cells. GGT5 is also involved in the process of cell adhesion and cell‐matrix adhesion, which is important for the colonization and growth of tumor cells in the microenvironment. Higher GGT5 expression may be associated with poorer clinical outcomes, such as increased risk of tumor recurrence and metastasis and reduced survival. Therefore, GGT5 is expected to be a potential biomarker for the diagnosis and prognosis of OSCC, as well as a potential target for therapy [64].
	Hepatocellular carcinoma (HCC)	GGT6 was differentially expressed between hepatocellular carcinoma primary samples and normal control tissues. There was a positive correlation between GGT6 and GGT5, and the expression of GGT6 was less correlated with the overall survival (OS) and disease‐free survival (DFS) of HCC patients. The DNA methylation level of GGT6 was significantly higher in normal tissues than that of the corresponding HCC samples and a negative correlation between the DNA methylation and mRNA expression was observed. GGT6 expression was positively correlated with the infiltration level of CD4^+^ T cells, macrophages, neutrophils and dendritic cells in HCC. The expression of GGT6 was positively correlated with the infiltration levels of CD4^+^ T cells, macrophages, neutrophils and dendritic cells in HCC [15].
	Clear cell renal cell carcinoma (ccRCC)	GGT6, a component of metabolism related prognostic profile (MRPS), is expressed at low levels in renal clear cell carcinoma tissues compared to normal tissues, and samples with high expression levels of GGT6 showed a significant increase in the overall survival (OS) of patients compared to samples with low expression levels. Zhou, R et al. found that knockdown of GGT6 significantly promotes renal cancer cell proliferation, migration and invasion ability, GGT6 may have an inhibitory effect on the occurrence and development of ccRCC and is expected to be a therapeutic target of ccRcc [65].
	Papillary renal cell carcinoma (pRCC)	The expression level of GGT6 in papillary renal cell carcinoma (pRCC) tumor samples was lower than that in normal samples, and DNA methylation and gene expression survival analyses revealed that the DNA methylation level of GGT6 was altered in pRCC, and that the elevated methylation level could have suppressed the expression of the GGT6 gene, which was correlated with the lower survival rate of patients. Since GGT6 is coexpressed with HHLA2, which plays a role in immune escape, it is hypothesized that GGT6 may also be associated with immune escape from tumors, which requires further experimental studies to verify [19].
	Low‐grade Glioma (LGG)	GGT6 expression is downregulated in low‐grade gliomas (LGG), which has significant prognostic value and can be used as a potential early detection biomarker. In combination with KRT33B and KRT75, it can improve the prediction of LGG, glioblastoma (GBM) and LGG to GBM transformed tumors [41].
	Papilloma of the choroid plexus	Comparative genomic hybridization (array CGH) analysis of choroid plexus papilloma DNA arrays by de León et al. reveals amplification of SPNS2, GGT6, SMTNL2, PELP1, MYBBP1A, and ALOX15 genes in chromosome 17p [66].
	Thyroid cancer (TC)	Li et al. found that the differentially expressed GGT6 gene could be one of the key genes in metabolism related prognostic model of thyroid cancer in thyroid cancer tissues by differential expression analysis, Cox regression and LASSO analysis. Together with AWAT2, ENTPD1, PAPSS2, CYP26A, ACY3, and PLA2G10 metabolism related genes, GGT6 gene constitutes a 7‐gene prognostic model, which can be used as an independent predictor to effectively predict the prognosis of TC in thyroid cancer as well as to provide guidance for clinical treatment [67].
	Gastric cancer (GC)	GGT6 gene expression is upregulated during AMK‐induced gastric cancer formation, which may provide favorable conditions for the proliferation and survival of gastric cancer cells, and GGT6, together with other genes, such as Mapk13, Nme1, Gsta4, etc., participates in the signaling and gene regulatory network during AMK‐induced gastric cancer formation. GGT6 may be used as a potential biomarker or a therapeutic target that provides new ideas for early diagnosis and treatment of gastric cancer) [68].
	Colon	In colon cancer tissues, the expression of GGT6 was significantly lower than that in paracancerous tissues, suggesting that it may play an important role in the development of colon cancer. After treating colon cancer cell line HCT116 by Nutlin, Liu Zichao et al. found that GGT6 gene expression was significantly upregulated and its mRNA level was positively correlated with p53 protein expression. Further dual luciferase reporter assays also verified the binding regulatory effect of p53 on the promoter region of GGT6. activation of the p53 signaling pathway affects biological processes such as the cell cycle, DNA replication and chromosome segregation, and GGT6, as a downstream target gene of p53, may be involved in the regulation of these processes, which in turn affects the biological behaviors of colon cancer cells. tCGA‐COAD Data analysis showed that GGT6 expression was higher in colon cancer tissues than in paracancerous tissues, and the overall survival time was longer with high GGT6 expression than with low GGT6 expression, which was hypothesized to be a cancer inhibitory factor in colon cancer, and its expression level could be used as a potential biomarker for patients' prognosis. GGT has an important role in the synthesis of glutathione, which plays a key role in cellular antioxidant defense. GGT6 may affect the tumor microenvironment by regulating the intracellular oxidative stress state, which in turn has an impact on colon cancer progression [69].
GGT7	Glioblastoma multiforme (GBM)	Glioblastoma (GBM) is the most malignant primary brain tumor in adults, with a median survival time of one and a half years. GGT7 is a novel GGT family member. GGT7 has an approximately 20‐fold higher mRNA expression in the brain than in other normal tissues and a lower expression in gliomas than in normal brain. It is a novel regulator of glioblastoma growth. GGT7 induces GBM development and growth through the regulation of Antioxidant damage (ROS) induces the development and growth of GBM, and GBM with low expression of GGT7 has a poorer prognosis, GGT7 may be a promising prognostic biomarker and potential therapeutic target for GBM [20].
	Hepatocellular carcinoma (HCC)	The expression of GGT7 in hepatocellular carcinoma (HCC) tissues was higher than that in normal liver tissues, and the overall survival (OS) of HCC patients with high expression of GGT7 mRNA was poorer, and the disease‐free survival (DFS) was shorter, which was correlated with a poor prognosis. GGT7 regulated the tumor immune microenvironment, and was associated with the infiltration level of immune cells such as CD8^+^ T cells, CD4^+^ T cells, macrophages, and dendritic cells associated with the infiltration level of immune cells such as CD8^+^ T cells, CD8^+^ T cells, macrophages and dendritic cells, affecting tumor progression and immune escape mechanisms. The methylation level of the CpG site in the promoter region of GGT7 was negatively correlated with gene expression, and the DNA methylation of GGT7 was higher in the normal tissues than in the corresponding HCC samples. This altered methylation status may lead to aberrant expression of GGT7 in tumor tissues. GGT7 is expected to serve as a biomarker for prognostic assessment of HCC and provide new targets for future therapeutic strategies [15].
	Gastric cancer (GC)	GGT7 expression was suppressed in gastric cancer through promoter methylation, and higher levels of GGT7 expression in adjacent nontumor tissues were significantly associated with favorable survival of gastric cancer patients as an independent prognostic factor. GGT7 was shown to significantly inhibit the growth, G1‐S phase transition, and migration and invasive ability of gastric cancer cells, and significantly inhibited the growth of subcutaneous xenograft tumors and lung metastasis in a nude mouse model. GGT7 directly interacts with RAB7, a key protein regulating mitochondrial autophagy, and GGT7 increases mitochondrial autophagy by recruiting RAB7 and inducing its nuclear‐to‐cytoplasmic GGT7 directly interacts with RAB7, a key protein that regulates mitochondrial autophagy, by recruiting RAB7 and promoting its translocation from the nucleus to the cytoplasm, GGT7 increases mitochondrial autophagy mediator/inducer and induces mitochondrial autophagy, which inhibits intracellular reactive oxygen species (ROS) levels, and in turn, the MAPK signaling pathway, and inhibits the development of gastric cancer. Expression of GGT7 in adjacent nontumor tissues may be used as a prognostic biomarker of gastric cancer patients [70].
	Pancreatic ductal adenocarcinoma (PAAD)	It was found that the expression of GGT7 was upregulated in MIA PaCa‐2 and PANC‐1 cells, and overexpression of GGT7 promoted the proliferation and migration of pancreatic cancer cells. The STRING database predicts that TXNDC12 activates GGT7 through protein–protein interactions with GGT7, and that GGT7 influences the iron death of PAAD cells through the regulation of GSH metabolism. which in turn promotes the development of pancreatic ductal adenocarcinoma [10].
	Breast cancer (BC)	Univariate analysis of breast cancer patients with low GGT7 expression have poor prognosis and high risk of recurrence and metastasis, and are potential breast cancer metastasis suppressor genes. Changes in the expression level of GGT7 may affect the malignant progression of tumor cells, and correlate with the clinical stage and the number of positive lymph nodes, and the detection of the expression level of GGT7 can accurately assess the prognosis of breast cancer patients, and provide an objective basis for the formulation of individualized clinical treatment plans [71].
GGTLC1	Endometrial cancer (EC)	GGTLC1 is a protein‐coding gene initially named GGTL6, later renamed GGTLA4, and changed to GGTLC1 in 2008. It has been found that GGTLC1 is highly expressed in endometrial cancers, and this high expression negatively correlates with immune cell infiltration and DNA methylation, and promotes tumor progression by regulating the TGF‐β/Smad signaling pathway. Knockdown of GGTLC1 inhibited the proliferation and migration of endometrial cancer cells and induced cell cycle arrest and apoptosis. GGTLC1 may be a promising target for endometrial cancer therapy [72].
	Prostate cancer (PC)	In prostate cancer tissues, the expression level of GGTLC1 was significantly higher than that in normal prostate tissues. The high expression of GGTLC1 may promote tumor cell proliferation and survival by enhancing the antioxidant capacity of the cells, enabling prostate cancer cells to better cope with oxidative stress. In addition, GGTLC1 may be involved in regulating intercellular signaling in the tumor microenvironment by affecting the remodeling of the extracellular matrix and the expression of cell adhesion molecules, which promotes tumor migration and invasion. GGTLC1 may also interact with tumor‐associated fibroblasts (CAFs) and immune cells to regulate the immunosuppressive properties of the tumor microenvironment, which in turn favors tumor progression. Since high expression of GGTLC1 in prostate cancer tissues correlates with tumor malignancy and poor prognosis, it can be used as a prognostic marker for prostate cancer. Silencing the expression of GGTLC1 by inhibiting its activity or expression, using small molecule inhibitors or utilizing gene editing techniques such as CRISPR/Cas9, may be a new therapeutic strategy for prostate cancer treatment [73].
	Non‐small cell lung cancer (NSCLC)	It was found that in the NSCLC tumor microenvironment, GGTLC1 expression was downregulated in tumor‐T cells compared with normal‐T cells, and the infiltration level of CD3^+^GGTLC1^+^ T cells was lower than that of the surrounding normal tissues in NSCLC tissues, and that GGTLC1 was able to inhibit ROS‐induced T‐cell senescence and reduce the production of senescence‐associated secreted phenotype (SASP) factors (e.g., IL6 IL6, IL8), thus inhibiting the invasion and migration of NSCLC cells, which in turn affects tumor progression and immune escape. NSCLC patients with high expression of GGTLC1 have a relatively better prognosis, and it is expected to be a therapeutic target for the treatment of NSCLC [74].
	Lung adenocarcinoma (LUAD)	Studies have shown that GGTLC1, together with five other novel neutrophil extracellular traps (NETs)‐associated genes (UPK1B, SFTA3, SCGB3A1, ABCC2, and NTS), may reflect a complex molecular regulatory network in the tumor microenvironment of LUAD, which synergistically affects tumor progression and prognosis. Mo et al. through a TCGA‐LUAD dataset screening analysis of the TCGA‐LUAD dataset, it was found that changes in the expression level of GGTLC1 were characterized in the high NETs score group compared to the low NETs score group. GGTLC1, as a component of the prognostic model, is involved in the computation of NETs scores after its expression is multiplied by the corresponding LASSO coefficients, and high‐risk patients (with high NETs scores) tend to show suppression of immune‐related processes, and the aberrant expression of Abnormal expression of GGTLC1 may alter the tumor immune microenvironment, which in turn affects the survival and prognosis of patients.GGTLC1 is expected to be a potential biomarker for the diagnosis, prognosis, and therapeutic monitoring of LUAD, and to provide a new target for precision medicine in the future [75].
	Breast cancer (BC)	GTLC1, as a light chain encoding gene, was validated by DU et al. through functional enrichment analysis, immune microenvironment analysis, and in vitro cellular assays, and found that PRC1, GGTLC1, and IRS1 may mediate breast cancer chemoresistance through drug efflux, cell cycle regulation, redox state regulation, and alteration of the immune microenvironment. Research and development of therapeutic strategies targeting GGTLC1 may provide new therapeutic options for breast cancer patients [76].

### 2.3 GGT mRNA

Previous studies have reported increased GGT mRNA levels and GGT activity in the liver in response to alcohol and carcinogen intake. Studies have shown that GGT mRNA levels and GGT gene expression are highly correlated in biological models of tumors. GGT mRNA is a product of GGT gene transcription, and changes in the expression levels and activity of GGT mRNA and the GGT gene are closely related to tumorigenesis and progression. The GGT gene has a complex structure, and its transcription is usually controlled by several promoters, which results in the production of several isoforms of GGT mRNA. The detection of human serum and tissue GGT mRNA is helpful for the early diagnosis of tumors and monitoring postoperative recurrence. The induction of GGT mRNA isoforms and the involvement of multiple signaling pathways vary depending on the cell type and stimulus. The GGT gene encodes at least the following mRNAs: (1) GGT I mRNA mainly encodes GGT in normal human tissues, and it is tissue specific. (2) GGT I mRNA with the apical structure removed, which encodes mainly the GGT light chain (1, 2 are transcription products of the same gene). (3) GGT II mRNA is expressed mainly in the liver. (4) GGT III mRNA is the transcription product of another gene whose top is removed. GGT III mRNA has several point mutations and an unsheared 81 bp intron in the open reading frame. GGT III mRNA is truncated; is tissue and pathology specific; and is present in the human placenta, sigmoid colon, lung, and 50% of acute lymphoblastic leukemia blood cells. It is not found in healthy lymphocytes [77]. Currently, GGT I mRNA, which has a tissue‐specific distribution in vivo and is found mainly in the pancreas, fetal liver, placenta, lung, and hepatocellular carcinoma tissue cells, is the focus of study. The GGT I mRNA open reading frames (ORFs) are the same in different tissues, and the main difference lies in the 5 NC regions, where at least eight exons are present. In different tissues, 5 NC exons can undergo selective or tissue‐specific variable splicing that regulates the expression of GGT I mRNA in different tissues. Researchers believe that at different stages of liver embryonic cell differentiation, GGT I gene expression is driven and regulated by different promoters and produces different mRNAs [78]. Han G refers to the different forms of GGT I mRNA in the human fetal liver, hepatocellular carcinoma tissues, and placenta as isoforms A, B, and C, respectively, and hepatocellular carcinoma occurrence is closely related to the conversion of GGT I mRNA from subtype A to subtype B. GGT I mRNA helps in the diagnosis of hepatocellular carcinoma [79]. GGT mRNA splice variants (e.g., isoform B in HCC) can be used as tissue‐specific diagnostic targets linking genetic alterations and enzymatic phenotypic differences. Daubeuf suggested that GGT mRNA in hepatocellular carcinoma cells is predominantly of subtype B. Sheen suggested that the GGT I mRNA subtype B is related to the serum AFP level in tumor foci, degree of differentiation of the tumor cells, residual postoperative infiltration, postoperative infiltration of the tumor, postoperative recurrence, and postoperative survival, and monitoring GGT I mRNA is an important indicator for predicting whether recurrence occurs after surgery. Moreover, Weidong Shen experimentally verified that the positive rate of the GGT I mRNA B subtype combined with AFP mRNA in diagnosing hepatocellular carcinoma could reach 98%, the positive rate of small hepatocellular carcinoma could reach 95.2%, and the combined monitoring of the GGT I mRNA subtype and AFP mRNA could aid in the early diagnosis of hepatocellular carcinoma [80]. In contrast, Korea Kyung reported that there was no correlation between serum AFP and peripheral blood GGT mRNA B isoforms, which is inconsistent with the findings of some studies [78]. Notably, the regulatory mechanisms of GGT mRNA and GGT genes are complex and involve a variety of factors, and their specific roles still need to be further investigated.

## 3 γ‐Glutamyl Transpeptidase (GGT)

**FIGURE 1:**
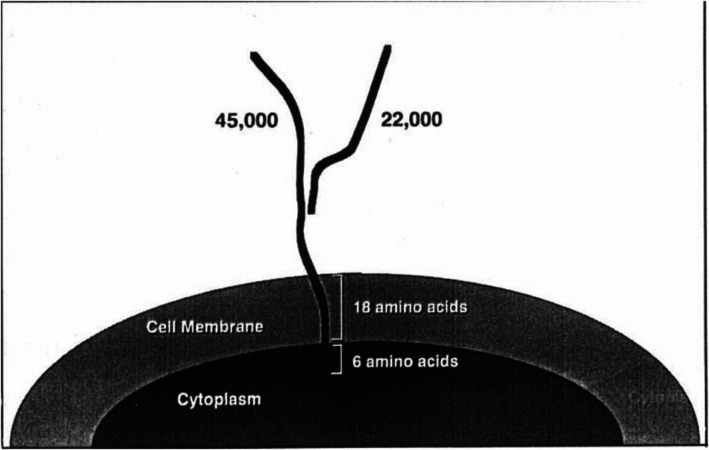
GGT membrane topology for extracellular glutathione hydrolysis [16].

In 1948, Binkley and Nakamura reported that rat kidneys contain an enzyme that initiates the hydrolysis of glutathione by severing the γ‐glutamyl bond of glutathione. γ‐Glutamyltransferase (GGT) is widely distributed throughout living organisms, including plants, yeast, and bacteria. Human GGT is encoded by a single gene that encodes a GGT‐like protein, and GGT mRNA carries all the genetic information needed to synthesize the GGT enzyme. The messenger RNA encoding the GGT enzyme directs ribosomes to be translated and synthesized into the GGT enzyme; however, it has not been demonstrated that mRNA expression is correlated with the enzyme's function. γ‐Glutamyltransferase (GGT, e.g., 2.3.3.2) is a plasma membrane‐bound sugar heterodimer whose degree of glycosylation varies across tissues, controls key redox sensitive functions, and regulates the balance between proliferation and apoptosis. γ‐Glutamyltransferase (GGT) belongs to the aspartyl N‐terminal nucleophilic hydrolase superfamily (Ntn), and the N‐terminal catalytic nucleophilic reagent is capable of cleaving amide bonds and possesses both γ‐glutamyl hydrolase and transketolase activities. The expression of GGT in cysteine homeostasis is crucial, and its induction has been linked to the pathology of asthma, reperfusion injury, and cancer [81]. GGT is anchored to the cell membrane as an extracellular surface enzyme, with the active site pointing to the extracellular space (Figure 1). GGT is usually expressed in epithelia and is found predominantly in neutrophils [82, 83], macrophages, and platelets [84], as well as in the luminal surfaces of glands and duct lining with secretory or absorptive‐competent, protein‐rich epithelial cells, with the highest GGT activity on the surface of the luminal mesenchymal cells of the proximal tubule of the kidney (glutathione is not retained by the glomerulus and therefore must be reabsorbed as it passes through the tubule). The inhibition of GGT leads to high urinary excretion of intact glutathione. The inhibition of GGT leads to increased urinary excretion of intact glutathione. However, serum levels are low, and serum GGT activated enzymes are derived mainly from hepatic capillary cholangiocytes and hepatic Kupffer's cell microsomes, followed by ducts within the pancreatic follicular villi, the apical surfaces of the intestinal crypt endothelial cells, the epithelial luminal surfaces of the ducts of the genitourinary system (prostate gland and testicular tubules), the ducts of the sweat glands and salivary gland ducts, and the surfaces of the endothelial cells of the capillaries in the nervous system (molecular weights of GGT in purified form vary slightly between tissues), and in embryonic stages of development. (The molecular weight of purified GGT varies slightly from tissue to tissue) and is most abundant in the liver during the embryonic period, when GGT is highly active and rapidly decreases to low levels after birth. Small bands of positively stained stromal cells and GGT positive histiocytes were also observed in some tissues. Analysis of human fetal tissues revealed that GGT is differentially expressed developmentally between humans and rodents. These findings provide a basis for further studies on GGT induction in tumors and the effect of GGT expression on the tumor chemotherapeutic response [85]. GGT is a marker of glutathione (GSH) depletion in the liver and regulates the metabolism of the most abundant intracellular antioxidant, thiol glutathione (GSH). GGT plays a key role in the control of redox homeostasis. It hydrolyzes extracellular GSH and provides cells with cysteine, which is required for intracellular GSH and protein biosynthesis. Elevated GGT reflects increased exposure to organic exogenous compounds metabolized via glutathionylation in the liver; thus, the upregulation of GGT endows cells with greater antioxidant capacity and the advantage of rapid cellular growth. GGT has been implicated in biotransformation, nucleic acid metabolism, and oncogenesis. GGT is upregulated during inflammation and the development of various human tumors, and it is also involved in many physiological disorders related to oxidative stress [74]. GGT converts leukotriene C4 (LTC4) to leukotriene D4 (LTD4), which, as the most potent cysteinyl leukotriene among the chemokines in inflammatory cells, is directly involved in the regulation of inflammatory processes and promotes cell proliferation and differentiation. When the genes controlling GGT synthesis are out of control, embryonic type GGTs with different configurations and physicochemical properties are secreted, and GGTs are abnormally expressed in tumor tissues, which affects the occurrence and development of malignant tumors. Therefore, understanding GGT and its role in tumors is useful for the early diagnosis of tumors as well as for the development of therapeutic interventions that more effectively target human GGT.

### 3.1 GGT Molecular Structure

**FIGURE 2:**
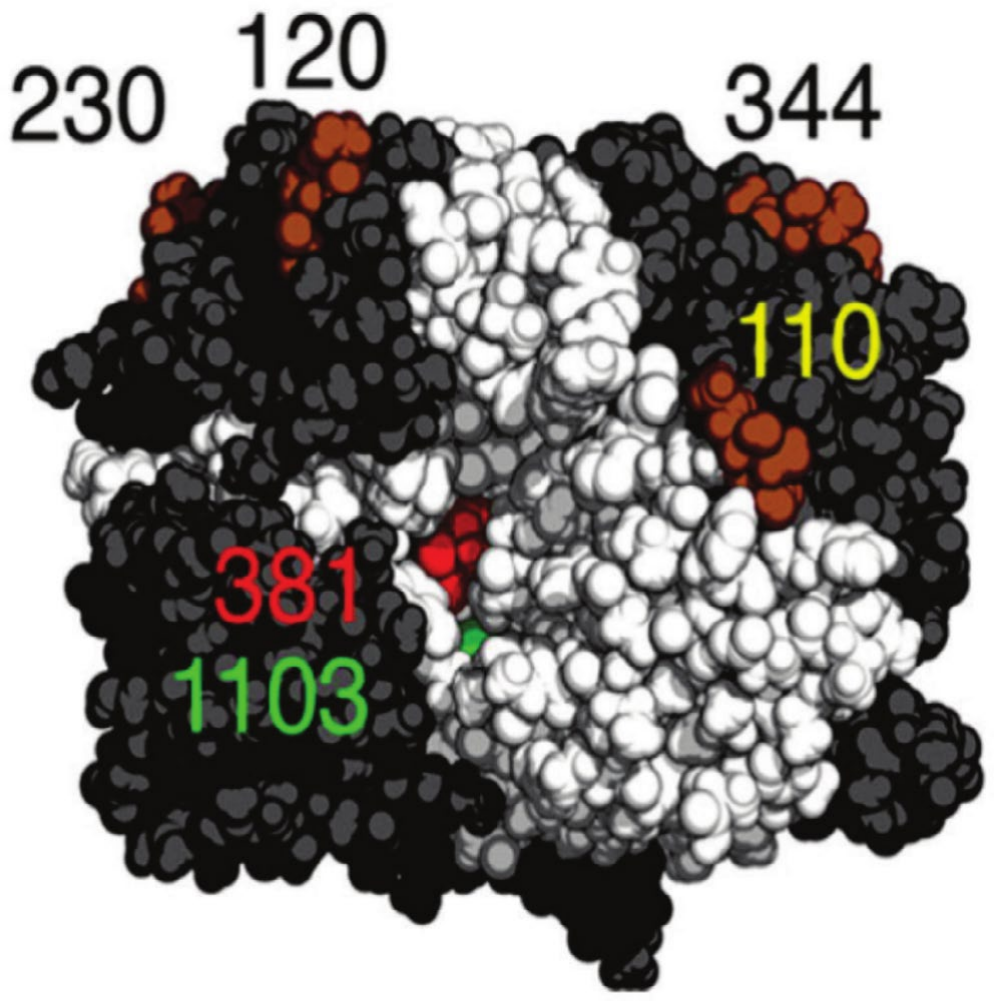
The van der Waals surface of GGT highlighting the active site cleft and key functional residues.

**FIGURE 3:**
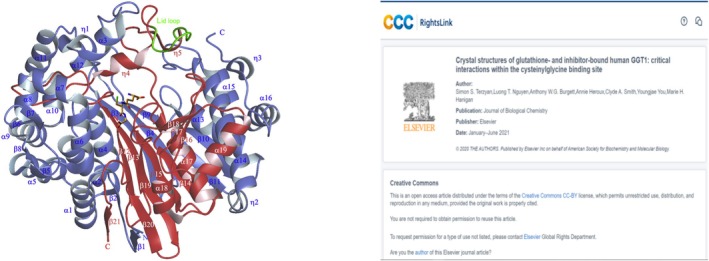
Ribbon presentation of GGT with bound GSH substrate.

GGT is a glycosylated heterodimeric protein encoded by a single gene, and the degree of glycosylation varies in different tissues, resulting in different molecular masses. Human GGT has seven N‐glycosylation sites (Figure 2), glycosylation is required for zymogen maturation, and the altered N‐glycosylation pattern of GGT in malignant tissues can be used for early tumor detection. GGT is synthesized as two inactive transmembrane polypeptides or zymogens as membrane‐bound glycoproteins, which are translated to form a heavy chain and a light chain and can be autocleaved into amino acid residues containing amino acid residues 1–380 large subunits and 381–569 small subunits. The amino acids of both the large and small subunits are present in the active site of the enzyme (Figure 3). (1) Heavy chain: The heavy chain has a relative molecular mass of 48–66 kDa and is composed of 351 amino acid residues. The heavy chain is preceded by a single peptide chain (signal peptide) composed of 27 amino acid residues, which is fat soluble. The heavy chain has a single transmembrane structural domain, as well as an extracellular component that binds the light chain. The heterodimeric GGT is anchored to the cytoplasmic membrane by the transmembrane fragment at the N‐terminal end of the large subunit of the heavy chain, and there are six potential N‐glycosylation sites on the large subunit. The N‐terminal (residues 5–26) structure of the large subunit is a hydrophobic single pass transmembrane structural domain, and the heavy chain is hydrophobic. (2) Light chain: relative molecular mass 22–30 kDa, composed of 189 amino acid residues; the primary structure of the small subunit is more conserved than that of the large subunit, containing a glycosylation site and most of the residues forming the enzyme active site (including the catalytic threonine, which is responsible for autocatalytic cleavage and enzyme activity); the light chain has hydrophilic properties, and the light chain is located outside the cell and performs the catalytic activity of the catalytic enzyme. Contiguous regions: a well‐characterized donor site that specifies the substrate for donor γ‐glutamyl binding and an acceptor site where glutathione hydrolysis occurs in the extracellular space via a ping‐pong mechanism [1]. Thr‐381 is the N‐terminus of the small subunit, and the highly conserved Thr‐381 favors protein hydrolysis cleavage as a nucleophilic reagent, with hydroxyl attack on the preceding residue by the hydroxyl group of Thr‐381 in the proenzyme (glutamate Gly380 in GGT) to the carbonyl group, forming a tetrahedral intermediate (γ‐glutaminase complex) stabilized by two conserved glycines (Gly473, Gly474), which is cleaved to yield a new N‐terminal residue [81]. Localization of the donor substrate in the active site was stabilized by hydrogen bonding between glutamate and key neighboring residues (Arg107, Ser451, Ser452, and Asn401). Upon hydrolysis, the cysteine glycine dipeptide is released and cleaved into cysteine and glycine by cell surface dipeptidases, while the outwardly directed γ‐glutamyl group can be transferred to a second substrate (acceptor), binding to the acceptor site via the conserved residues Lys562 and Tyr403. GGT is synthesized and cleaved via a catalytic reaction in which GGT has nonphosphate‐dependent glutaminase activity; that is, it has a glutamine binding site. The crystal structure of glutamate‐bound mature GGT shows that the large and small subunits are tightly intertwined and interact with each other in a conserved “sandwich‐like” three‐dimensional structure consisting of four layers of stacked αββα folds and that their interactions are critical for enzyme activity [86].

The van der Waals surface representation shows the large subunit (dark gray) and small subunit (white). The catalytic Thr‐381 (red) is positioned in the deepest region of the small subunit cleft. Four of the seven potential N‐glycosylation sites (Asn‐110, −120, −230, and−344) are visible in this orientation, with their basal N‐acetylglucosamine (GlcNAc) residues depicted as dark orange spheres. An anion‐binding site (green) within the cleft is labeled (site 1103) [87].

The large subunit is shown in blue, and the small subunit is in red. The lid loop region (small subunit) is highlighted in green. GSH is depicted as sticks with carbon atoms in orange, oxygen in red, nitrogen in blue, and sulfur in green. Active site localization is indicated by GSH placement [86].

### 3.2 GGT Promotes Tumorigenesis and Progression

Large prospective studies have revealed abnormally elevated levels of GGT expression in a variety of human malignant tumor tissues, sera, and serum isolated from head and neck squamous cell carcinomas [88, 89]; squamous cell carcinoma of the esophagus; laryngeal carcinoma; thyroid carcinoma; melanoma [84]; cholangiocarcinoma; lung carcinoma; gastric cancer [90]; pancreatic cancer; hepatocellular carcinoma; intrahepatic cholangiocarcinoma [91]; colorectal carcinoma [92]; rectal adenomas [93]; prostate cancer; breast cancer [94, 95, 96]; cervical carcinomas; ovarian carcinomas; endometrial carcinomas [97]; lymphoma [98]; and many other human malignancies with abnormally elevated levels of GGT expression in tissues, serum, and exosomes isolated from serum; however, some renal cell carcinoma [19, 21, 22, 59, 60, 65], glioblastoma [20, 41], and breast cancer (BRC) [57, 71] tissues exhibit GGT loss. On multivariate analysis, GGT was found to be a possible prognostic indicator for early preoperative non‐small cell lung cancer [99], bladder cancer (BLC) [100, 101], and postoperative recurrence of confined colorectal cancer liver metastases (CRCLM) [102], and the CA199/GGT ratio could be used as a prognostic indicator for juxtapapillary carcinomas, pancreatic ductal adenocarcinomas (PDACs) [103], pancreatic head carcinomas [104] and pancreatic carcinoma (PA) [105], and biliary obstruction of distal cholangiocarcinoma (DCC) [106] tumor recurrence, overall survival (OS), cancer‐specific survival (CSS), disease‐free survival (DFS), and long‐term poor clinical prognostic predictors; high baseline serum ALP and GGT were associated with liver metastases in colorectal cancer (CRC) [107], and pancreatic cancer hepatic metastasis treated with nab‐paclitaxel/gemcitabine [108] and metastatic renal cell carcinoma (MRCC) [109, 110] receiving nivolumab targeted therapy, which is a convenient, rapid, and cost‐effective means of early prediction of anti‐tumor treatment efficacy; GGT in combination with CEA predicts that metastatic colorectal cancer (mCRC) overall survival (OS) and progression‐free survival (PFS) play an independent role and can be a better prognostic predictor [111]. In addition, several cohort studies have further explored the associations between serum GGT levels and cancer specific mortality and have attempted to control for potential confounders. Although several studies have suggested that GGT may have independent prognostic value, its association with cancer mortality may still be influenced by confounding factors such as alcohol intake, obesity, and nonalcoholic fatty liver disease (NAFLD). For example, the UK Biobank study revealed that the association between GGT and lung cancer was more pronounced in obese people, smokers, and habitual alcohol drinkers, suggesting that these variables may enhance the prognostic impact of GGT [112]. A Chinese cohort study of patients with pancreatic ductal adenocarcinoma also revealed that GGT can be used as a potential prognostic indicator for metastatic pancreatic cancer, adjusting for age, chemotherapy status, fasting glucose, and albumin, but its prognostic value may also be affected by metabolic factors such as alcohol, obesity, and NAFLD [113]. In addition, a Korean national study revealed that the association between GGT and cancer mortality was more prominent in alcohol drinkers than in those with a history of cancer [2]. These findings suggest that elevated GGT may partially reflect underlying metabolic disorders or liver dysfunction rather than a direct carcinogenic effect. Therefore, in future studies, the control of the following variables needs to be further strengthened: (1) Alcohol intake and frequency: alcohol is one of the main external factors of elevated GGT and needs to be precisely quantified. (2) Obesity and metabolic syndrome, especially BMI, waist circumference, insulin resistance and other indicators. (3) Nonalcoholic fatty liver disease (NAFLD): NAFLD should be recognized by imaging or serological indicators. (4) Liver function related indicators, such as ALT, AST, and ALP, are used to exclude the interference of liver disease. In addition, subgroup analysis should be further refined to identify the independent prognostic value of GGT in different populations. For example, whether GGT can predict cancer death in individuals without liver disease or metabolic syndrome is an important direction for future research. In summary, despite the prognostic potential of GGT as a marker of oxidative stress and inflammation, its independent role in cancer mortality still needs to be validated under the control of more stringent confounders. Future studies should employ multivariate adjustment models, propensity score matching, or Mendelian randomization to improve the reliability of causal inference [114, 115, 116].

### 3.3 Diagnostic Significance of GGT in Hepatocellular Carcinoma

In this study, Fei Zhu and colleagues first applied the propensity score matching method to rigorously balance the baseline data of the high GGT group and the normal control group, covering several key variables, such as sex, serum ALB, glutamate aminotransferase, macrovascular invasion, maximum tumor diameter, and tumor number, to reduce the potential interference of confounding factors. On this basis, Cox multifactorial analysis revealed that GGT could not only be used to assess the liver function of patients with hepatocellular carcinoma (HCC) effectively but also play an important role in predicting postoperative tumor recurrence and long‐term survival. The results of this study revealed that the independent risk factors affecting the overall survival (OS) of HCC patients included GGT > 50 U/L, macrovascular invasion, tumor diameter ≥ 10 cm, and number of tumors ≥ 3, among which the risk ratio (HR) for GGT > 50 U/L was as high as 1.78, with 95% confidence intervals (CIs) ranging from 1.26 to 2.50 and a *p* value of 0.001, which indicated that GGT > 50 U/L was associated with the overall survival rate of patients. This finding indicates that it is an important risk factor that should not be ignored because of its strong association with postoperative overall survival. Among the independent risk factors affecting the postoperative tumor‐free survival (RFS) of HCC patients, in addition to the above factors, an AFP ≥ 400 ng/mL and a GGT > 50 U/L were also included, which corresponded to an HR of 1.430, a 95% CI of 1.08–1.90, and a *p* value of 0.013, once again highlighting the unique value of the level of GGT in the prediction of the risk of tumor recurrence [117]. Hepatocellular carcinoma (HCC) is a chronic inflammatory disease caused by hepatitis B virus (HBV) and hepatitis C virus (HCV) infection, chemical toxicity, or nonalcoholic fatty liver disease. Serum GGT activity is a rapid, reliable, and cost‐effective method for assessing liver function. The significance of baseline serum GGT in the diagnosis of hepatocellular carcinoma is as follows: serum GGT > 200 U/L can predict the risk of hepatocellular carcinoma in patients with hepatitis B and has some significance in the suspicion of early hepatocellular carcinoma; serum GGT > 450 U/L and serum GGT are significantly greater in primary hepatocellular carcinoma patients than in cirrhosis patients, which is important for the diagnosis of hepatocellular carcinoma. Elevated GGT is correlated with early hepatocellular carcinoma (HCC) size and poor prognosis. Independent of alpha‐fetoprotein AFP. Zhang et al. reported that a high serum GGT concentration was positively correlated with advanced TNM stage and tumor size and was an independent predictor of the overall survival (OS) rate in patients with primary HCC. Wang et al. reported that the serum GGT level was also an independent risk indicator for poor overall survival (OS) in patients with AFP‐negative HCC. GGT not only contributes to the diagnosis and prognosis of HCC patients but also may be a target for HCC immunotherapy [112, 118]. Serum GGT in combination with other tumor markers, such as AFP, PIVKA‐II, GP73, and the GGT/AST ratio, can improve the diagnostic sensitivity of HCC, especially in the early stage, small volume individuals with good liver function, and GGT may also reflect mitochondrial dysfunction after HCV eradication, which is a risk factor for the development of hepatocellular carcinoma [113]. High serum GGT expression is an important prognostic factor for poor overall survival (OS) and recurrence‐free survival (RFS) in advanced hepatocellular carcinoma (HCC) patients undergoing radical hepatectomy for HCC [119], as well as in advanced hepatocellular carcinoma (HCC) patients undergoing transcatheter arterial chemoembolization (TACE) [120]. GGT expression seems to be correlated with HCC invasiveness characteristics, tumor size range, and severity and is not related to disease‐free survival (DFS). A meta‐analysis of primary HCC patients revealed that a preoperative serum GGT concentration ≥ 38 U/L independently predicts the risk of recurrence and all‐cause mortality in patients with HBV‐associated HCC who are undergoing surgical resection (especially patients with tumors > 5 cm and who are treated with liver transplantation) and is a cost‐effective prognostic tumor biomarker for HCC resection, especially when the AFP concentration is ≤ 200 ng/mL in hepatocellular carcinoma patients [121]. GGT/albumin (GAR) is most helpful for prognosis and reflects significant differences in the aggressive characteristics of hepatocellular carcinoma [122]. If the GAR is high in nonmicrovascular hepatocellular carcinoma [123], intrahepatic cholangiocarcinoma (ICC) after hepatic resection [124], overall survival (OS) and disease‐free survival (DFS) are poor, and if a pretransplantation GAR ≥ 2.04 is associated with HCC liver transplantation (LT), prognostic and survival outcomes are independently correlated and could be used as prognostic indicators for mortality and tumor recurrence after LT [125]. The nomogram for the albumin/fibrinogen ratio (AFR)‐GGT/Plt (GPR) risk stratification is more reliable, convenient, and accurate for the prognostic prediction of HCC [126]. A ≥ 2‐fold increase in GGT versus ALP is the most important factor in transcatheter arterial chemoembolization (TACE) after surgery and is a predictor of bile duct injury [127]. The GGT/ALT ratio is a potentially effective predictor of vascular invasion and prognosis in patients with HBV‐associated HCC [128]. In addition, pretreatment serum GGT/Plt (GPR) has good sensitivity, accuracy, and prognostic value in HCC patients [129, 130] (Table 3).

**TABLE 3:** Value of GGT and related indicators in HCC prognosis.

Prognostic indicators	Study cohorts	Survival analysis results	Results of multifactor analysis	References
GGT > 50 U/L	432 patients with radical resection	5 year OS: 43.3% vs. 66.1%	OS:HR = 1.87 (1.07–3.26)	[117]
GGT > 90.5 U/L (TACE)	608 TACE treated patients	3 year OS: 8.6% vs. 27.9%	OS:HR = 2.021, *p* < 0.001	[120]
GAR > 333.33	335 patients without microvascular invasion	3 year PFS: 18.7% vs. 36.1%	PFS:HR = 1.80 (1.24–2.60)	[123]
GGT ≥ 60 U/L (HBV‐HCC)	330 cases of HBV related HCC	Significantly higher risk of recurrence	Recurrence: HR = 2.15, *p* < 0.001	[131]

## 4 γ‐GGT Isozyme

**FIGURE 4:**
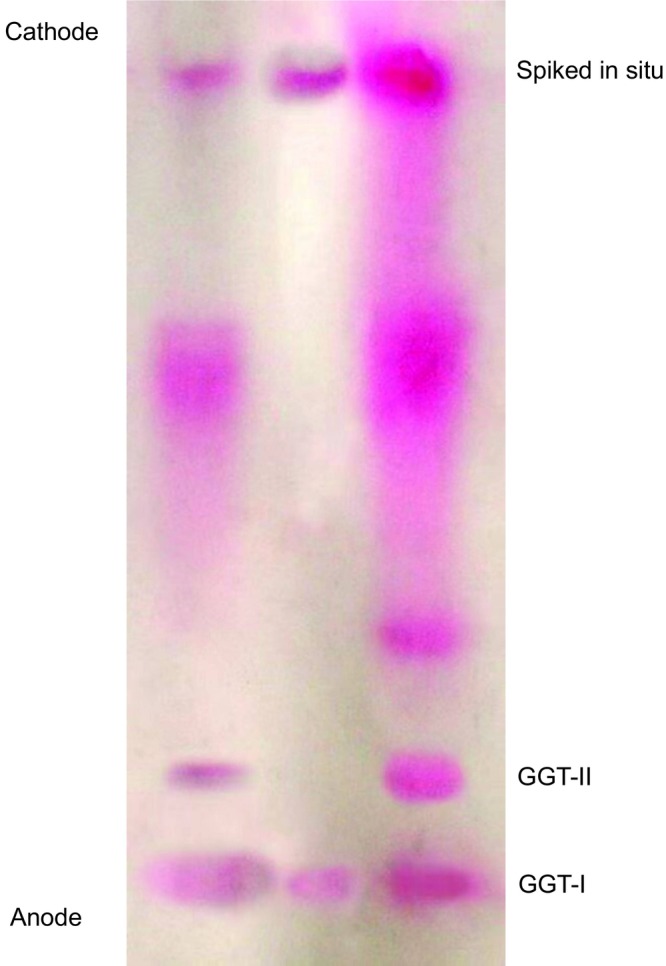
Serum GGT discontinuous gradient SDS‐PAGE results [134].

Yao Dengfu, Huang Jiefei et al. separated serum GGT by discontinuous gradient polyacrylamide gel electrophoresis (PAGE) on the basis of the different structures of γ‐GGT sugar chains and different mobilities and separated serum γ‐GGT into 11–13 bands, such as I, I′, II, III, IV, V, VI, VIIa, VIIb, VIIIa, VIIIb, and VIIIc, etc., and under normal physiological conditions, only some of the bands appeared [78]. The formation of γ‐GGT isozymes varies with the amount of sialic acid on their sugar chains; the species, and the antenna structure of the glycan chain is altered (malignant tumor tissues contain a significantly higher concentration of salivary acid than normal tissues do, which can lead to a low PI of the enzyme protein). The main enzymatic properties of GGT isozymes from different tissues are similar (e.g., km value, optimal pH, thermal stability, competitive inhibition of GSH), and the main differences are their electrophoretic ability and binding ability to lectins (Con A, WGA, RCA, UEA, etc.). The structure of the GGT sugar chain produced by hepatocellular carcinoma cells is altered, and this difference can appear in the pre‐and early stages of hepatocellular carcinoma, but the dynamic pattern of this change has not been fully clarified. The GGT‐II band (similar to the embryonic type GGT band) is the HCC‐specific band of hepatocellular carcinoma; the activity is significantly elevated in the serum of patients with hepatocellular carcinoma, and its sensitivity and specificity are superior to those of the total GGT level. Moreover, GGT‐II activity is positively correlated with the clinical stage of hepatocellular carcinoma (e.g., tumor size and metastasis), which may be related to the increased invasiveness of hepatocellular carcinoma cells and metabolic abnormalities and is potentially valuable in the assessment of tumor progression and prognosis [132, 133] (Figure 4, Table 4). This zone is not observed in other hepatobiliary diseases (metastatic cirrhosis, alcoholic hepatitis, and cholangiocellular carcinoma), which would help in the diagnosis and differential diagnosis of liver diseases. Clinical cohort analysis revealed that GGT‐II had a sensitivity of 78.7% and a specificity of 92.3% (AUC = 0.89) for the diagnosis of HCC and was superior to AFP (AUC = 0.67) in early stage disease and that hepatocellular carcinoma‐specific gamma‐glutamyltransferase isoform II (GGT‐II) is considered to be the best hepatocellular carcinoma biomarker, with the exception of alpha fetoprotein (AFP) [135] (Table 5). In addition, GGT‐II dynamics are significantly positively correlated with tumor load and portal vein invasion. The combination of GGT‐II with AFP and PIVKA‐II can increase the sensitivity of HCC detection to 95.2% [140], and the use of GGT‐II in combination with AFP for clinical diagnosis and population census of hepatocellular carcinoma patients will help improve the early diagnosis rate of hepatocellular carcinoma and enable more patients with hepatocellular carcinoma to be treated at an early stage. However, Qiu S reported that the pathogenesis of primary hepatocellular carcinoma (PHC) is complex and involves many factors and that GGT‐II is not a good indicator for early PHC but is more relevant to advanced PHC. GGT‐II is not a suitable marker for early PHC but can be used as an indicator for monitoring the treatment and recurrence of advanced viral hepatitis B‐related PHC [141]. Early diagnosis of PHC generally requires a combination of multiple markers, and the combination of AFP, α‐L‐fucosidase (AFU), PIVKA‐II, GPDA‐F, and GP73 can improve the positive detection rate of PHC [118, 140, 142, 143, 144, 145, 146, 147, 148], especially in AFP negative or low expression cases. This reflects that the clinical application of GGT‐II has not yet reached a broad consensus, and in the future, combining multiomics technology with the validation of its time window characteristics through large sample cohort studies is necessary to clarify its positioning in the precise diagnosis and treatment of liver cancer. GGT V is found mainly in colorectal cancer tissues (91.7%) and paracancerous tissues (72.7%), and the GGT V positivity rate is not correlated with Dukes' stage of colorectal cancer or with the degree of differentiation; moreover, its isoelectric point is consistent with the isoelectric point of placental type GGT (PGGT V). These results suggest that GGT V is an oncoprotein in colorectal cancer tissues and is expected to be a biochemical marker for the early diagnosis of colorectal cancer and monitoring of precancerous lesions [149]. Although GGT isozymes show potential diagnostic and prognostic value in disease diagnosis, they still face many challenges in the clinic. Current evidence comes from single center studies, and prospective multicenter trials are needed to validate standardized methods for detecting GGT isozymes on the basis of histopathology and to establish uniform operation procedures, quality control standards, and critical values. The role of GGT isozymes in clinical diagnosis can be fully utilized to provide strong support for early detection, accurate diagnosis, and effective treatment of diseases (Table 6).

**TABLE 4:** Summary of cohort studies of serum GGT levels and cancer‐specific mortality.

Studies	Type of cancer	Classification of GGT levels	Adjusted HR (95% CI)	Control of confounding factors	Reference
UK Biobank study	Lung cancer	GGT > 26.3 U/L vs. ≤ 26.3 U/L	HR: 1.206 (1.121–1.297)	Adjusted for age, gender, BMI, smoking status, frequency of alcohol consumption, and liver function indicators	[112]
China Pancreatic Cancer cohort study	Pancreatic ductal adenocarcinoma (PDAC)	GGT > 48 U/L vs. ≤ 48 U/L	HR: 1.53 (1.19–1.97)	Adjustment for age, chemotherapy status, fasting glucose, albumin, and other factors	[113]
Korean National cohort study	Multiple cancers (predominantly lung cancer)	GGT high level vs. low level (tertile)	HR: 1.33 (1.32–1.34)	Variables adjusted for sex, age, smoking, alcohol consumption, history of cardiovascular disease (CVD), history of cancer, etc	[2]

**TABLE 5:** Summary of performance metrics of GGT‐II in HCC diagnosis.

Characteristics of the study		GGT‐II			AFP comparative reference values
Detection methods	Specific immunization membrane adsorption method	Polyacrylamide gel electrophoresis	Rocket immunoelectrophoresis	Enzyme linked immunosorbent assay (ELISA)	Chemiluminescence
Sample size (HCC/control)	312/573	187/569	61/56	39/119	
Sensitivities	85.26% (266/312)	94.1%	86.9% (53/61)	66.7% (28/40)	62.5%
Idiosyncrasy	95.81% (549/573)	96.13%	92.1% (56/62)	91.8% (112/122)	95%
Positive rate of small hepatocellular carcinoma (< 5 cm)	59.09% (13/22)	Unreported	Unreported	Unreported	29%–52%
AFP negative HCC positive rate	67.39% (31/46)	78.57% (AFP < 400 μg/L)	88.9% (16/18)	77.8% (14/18)	30%–40% [136]
References	[137]	[78]	[138]	[139]	

**TABLE 6:** Comparative quantitative analysis of GGT isoenzyme II (GGT‐II) in hepatocellular carcinoma (HCC) patients and healthy controls.

Strip	Groups	Relative expression intensity (optical density ratio)	Statistical Significance
GGT‐II	HCC	12.51 ± 8.85 U/L	↑↑ Significantly higher, ***p < 0.001
GGT‐II	Healthy control	0.06 ± 0.14 U/L	Baseline level

## 5 GGT Fraction

GGT isoform detection is highly important for the diagnosis and prognosis of liver diseases and other diseases, but GGT isozyme detection methods have not been standardized and usually detect only specific GGT isozymes and cannot comprehensively analyze all the isoforms. In recent years, studies have focused on GGT fractions, which are different isoforms of GGT formed by posttranslational modifications (e.g., glycosylation) in different tissues or pathological states. The analysis of GGT fractions improves the sensitivity and specificity of detecting GGT activity [150] and may be of specific significance in the diagnosis and prognosis of diseases. GGT has been shown to be a variety of GGT subfractions that are active as a sum. GGT circulates in the blood in at least four different molecular forms, which are categorized according to their molecular weights as large GGT (b‐GGT), medium GGT (m‐GGT), small GGT (s‐GGT), and free GGT (f‐GGT); their concentrations and ratios vary under different pathological conditions, and different patterns of plasma GGT fractions have been described in certain liver diseases. Genetic syndromes and significantly elevated plasma GGT are usually associated with cytotoxicity during liver disease [84]. GGT is usually released as b‐GGT, and s‐GGT and f‐GGT are associated with exosomal vesicles and may be produced in the extracellular environment of the liver in response to a combination of bile acids and papain [84, 151]. The purification of b‐GGT was carried out via ultracentrifugation. b‐GGT has a molecular weight of 2000 kDa and a density of 1.063–1.21 g/mL. Detergents convert b‐GGT to s‐GGT. m‐GGT and s‐GGT have molecular weights of 1000 and 200 kDa, respectively, and densities of 1.006–1.063 g/mL and 1.063–1.21 g/mL, respectively, are unaffected by deoxycholic acid. After papain treatment, GGT activity returns to the f‐GGT peak. f‐GGT has a molecular weight of 70 kDa and a density greater than 1.21 g/mL. Two peaks were found in the bile, with characteristics similar to those of the b‐GGT and f‐GGT fractions in plasma. Centrifugal sedimentation characteristics and immunogold electron microscopy data suggest that b‐GGT in bile and plasma consists of membrane microcapsules; m‐GGT and s‐GGT may consist of bile acid micelles, and f‐GGT represents the freely soluble form of the enzyme [152]. High‐performance liquid chromatography mass spectrometry (HPLC–MS) can be used to accurately separate and quantify GGT isomers of different molecular weights, providing detailed compositional information and facilitating the differentiation between types of liver diseases and other pathological states. The fractionation of serum GGT by high‐performance gel filtration liquid chromatography can be used for the differential diagnosis of alcoholic liver disease (ALD) and nonalcoholic fatty liver disease (NAFLD), in which the alcohol metabolite acetaldehyde induces an increase in the expression of GGT, with the highest s‐GGT/t‐GGT ratio and the lowest f‐GGT/t‐GGT ratio in alcoholic liver disease (ALD) [153]. The MELD score is used to assess the prognosis of patients with end‐stage liver disease, whereas cirrhosis s‐GGT has the greatest elevation and the median b‐GGT/s‐GGT ratio is lower than that of healthy controls, suggesting severe impairment of hepatic structural function, and the ratio is more diagnostically accurate than either s‐GGT or total GGT, even though total GGT is a sensitive biomarker of liver parenchymal rearrangement in patients with cirrhosis and hepatocellular carcinoma in both reference ranges, corresponding to a higher MELD score and poor prognosis [154]. Cancer cells release GGT in the same form as b‐GGT is released by inflammatory cells [151]. Therefore, the GGT isoform assay is highly valuable in clinical diagnostic and prognostic assessment and can be used to accurately determine the degree of liver disease types, such as distinguishing alcoholic liver disease from nonalcoholic fatty liver disease, and can also be used to monitor disease progression and therapeutic efficacy, such as in liver transplantation patients, as it can be used as an indicator to assess the recovery of grafted liver function and rejection reactions. Malignant pleural mesothelioma (MPM) is a cancer caused primarily by the inhalation of asbestos fibers and has an extremely long latency period and poor prognosis. Molecular exclusion chromatography revealed that GGT components play different and specific biochemical roles and that MPM has a specific GGT enzyme activity pattern, with increases in b‐GGT and m‐GGT activities and a decrease in f‐GGT activity. Combining b−/f‐GGT activity with serum mesothelin related protein (SMRP) may improve the diagnostic accuracy of malignant pleural mesothelioma (MPM) [155]. For clinical implementation, standardized GGT isoform quantification by mass spectrometry or affinity‐based platforms is needed. HPLC MS has high equipment costs, but its high sensitivity and high resolution enable rapid and accurate batch detection of quantitative GGT isoforms to improve diagnostic accuracy. Although the initial investment cost of the instrument is high, by optimizing the use of the process, simplifying the operation, and improving the efficiency of the assay, the cost of a single assay can be lower than that of traditional methods, resulting in a cost‐effectiveness threshold of < $50 per assay.

## 6 Conclusions

Early tumor screening and diagnosis are the keys to effective treatment and good prognosis. Reliable and specific tumor biomarkers are the keys to accurate screening and diagnosis of early‐stage tumors. GGT was first proposed to play a role in tumor formation in the 1980s, when it was found to be overexpressed in pretumor liver foci of chemically treated rats, and the expression of GGT could provide a selective growth advantage to the cells within the foci at the stage of cancer promotion. In 1974, New York physician Dr. Lum reported that serum GGT was several times the upper limit of normal in patients with biopsy‐confirmed metastatic hepatocellular carcinoma, pancreatic carcinoma, and gallbladder cancer. A recent Korean study monitored 1,662,087 Koreans for 17 years and reported a positive gradient in HR for cancer risk assessment associated with GGT quintiles. The highest quintile had a 6–7 fold greater risk of liver cancer similar to that reported in the large Austrian study by Kazemi‐Shirazi et al. GGT has an important and complex role in the development and progression of malignant tumors, and high levels of serum GGT significantly increase the overall incidence of cancer, and GGT has been shown to be a significant contributor to the development of cancer in the rest of the head and neck [88], digestive organs [156, 157, 158], respiratory/intrathoracic organs [159], genitourinary organs [100, 101, 160], breast and female reproductive organs [94, 95, 96, 97], lymphoid and hematopoietic cancer malignancies [98], and metastatic tumors, where aberrant GGT expression has been observed [161]. GGT is an important hepatic enzyme, and tumor cells express high levels of GGT across the entire cell membrane. GGT is involved in tumor oxidative stress, programmed cell death, and the regulation of intracellular glutathione levels. GGT plays an important role in chronic inflammation, tumor formation, and cell proliferation by degrading extracellular glutathione, providing cysteine for intracellular glutathione (GSH) synthesis and regeneration, increasing intracellular glutathione (GSH) levels, and inducing pro‐oxidative responses. High GGT levels are significantly associated with poor therapeutic response and prognosis to neoadjuvant chemotherapy (NAC), and high pretreatment serum GGT levels correlate with tumor stage, which can indicate early risk of tumor development. Overall survival (OS) and progression‐free survival (PFS) are significantly lower in patients with high GGT expression than in patients with normal GGT levels and can be used as independent prognostic indicators for poorer survival. GGT is a promising tumor biomarker and potential therapeutic target for specific types of tumors, such as hepatocellular carcinoma, renal carcinoma, prostate cancer, and gastric cancer.

## 7 Outlook

Owing to the pleiotropic nature of GGT, studies on members of the GGT encoding gene family in oncology are rapidly evolving to identify correlations between DNA polymorphisms in cancer patients, and these key differentially expressed gene constructs provide new insights into individual diagnostic treatments. GGT has been plagued by poor specificity as a tumor marker, with GGT elevated in a wide range of tumor tissues and peripheral blood manifestations and lacking tissue specificity. The existence of disease‐specific patterns is controversial, and more research is needed in terms of the correlation of test results with disease. Human GGT is a GGT‐like protein encoded by a single gene, and the GGT gene generates mRNA through transcription. GGT mRNA carries all the genetic information needed to synthesize the GGT enzyme, and the messenger RNA encoding the GGT enzyme guides ribosomes to be translated and synthesized into the GGT enzyme. The level of expression of the mRNA directly affects the activity of the GGT enzyme, which then affects its physiological function. Currently, according to related studies, changes in γ‐glutamyltransferase activity are associated with pretumor or toxicity status in the liver or kidney, and molecular mechanism studies have revealed that GGT7 affects tumor oxidative stress tolerance and promotes the epithelial‐mesenchymal transition (EMT) process in hepatocellular carcinoma cells by modulating glutathione metabolism, which may be promising biomarkers for the prediction of survival in HCC as well as new targets for intervention in tumor invasion and metastasis [15, 53, 54, 162]. Monitoring of GGT I mRNA B isoforms helps in the early diagnosis of hepatocellular carcinoma [79, 80]. The serum s‐GGT and GGT‐II isoforms show significant specific expression patterns in primary hepatocellular carcinoma (HCC) and are closely associated with hepatocellular carcinoma occurrence, progression, and clinical staging [132, 133, 135, 141]. Taken together, the available data suggest that the GGT7 genes, GGT I mRNA B isoforms, s‐GGT, and GGT‐II isoforms are potentially valuable for early diagnosis, prognostic assessment, and selection of therapeutic targets for HCC; however, the relationship between the GGT mRNAs and GGT genes is complex and regulated by a variety of factors, and there is a lack of validated protein coding activity of the GGT7 genes, GGT I mRNA B isoforms, s‐GGT, and GGT‐II isoforms, which have not yet been demonstrated to be related in terms of function and regulatory mechanisms. Further validation of their relevance and synergistic diagnostic efficacy is needed.

Future research needs to explore the following directions: (1) expand the clinical sample size and conduct multicenter cohort studies to clarify the dynamic change patterns of different GGT family members in different subtypes and stages of HCC; (2) combine single‐cell sequencing and spatial transcriptome technologies to resolve the cell‐specific expression patterns of the GGT7 genes and their regulatory networks in tumor microenvironments so that a spatial and temporal expression map of GGT family genes in heterogeneous microenvironments of hepatocellular carcinoma can be constructed in the future. In the future, we can construct spatiotemporal expression maps of GGT family genes in the heterogeneous microenvironment of liver cancer. (3) Proteomic and metabolomic technologies can be used to elucidate the mechanism of regulation of the metabolic phenotype of hepatocellular carcinoma through differences in the enzyme activities of the s‐GGT and GGT‐II isozymes, including their roles in extracellular matrix remodeling, cell adhesion, and signaling. These findings will contribute to a better understanding of its function in tumorigenesis and progression and provide a theoretical basis for the development of new therapeutic strategies. (4) Highly sensitive detection methods (e.g., exosome‐based liquid biopsy technology) should be developed to optimize the clinical application of existing markers. In addition, exploring the combined diagnostic strategy of GGT family molecules and traditional markers such as alpha fetoprotein (AFP) and PIVKA‐II may promote innovations in the precision diagnosis and treatment of HCC. Ultimately, through the deep integration of basic research and clinical translation, more groundbreaking biological targets for early screening and individualized treatment of hepatocellular carcinoma are expected to be identified.

## Author Contributions

**Fei Wang:** investigation (equal), resources (equal), visualization (equal), writing ‐ original draft (equal), writing ‐ review and editing (lead). **Jianshan Yang:** resources (equal), validation (equal), writing ‐ original draft (equal), writing ‐ review and editing (lead). **Feng Zhu:** validation (equal), writing ‐ original draft (equal), writing ‐ review and editing (lead). **Xuebing Xu:** conceptualization(equal), data curation (equal), visualization (equal), writing ‐ review and editing (equal). **Junpeng Zhao:** validation (equal), visualization (equal), writing ‐ review and editing (equal). **Xudong Xie:** visualization (equal), writing‐review and editing (equal). **Xuyang He:** data curation (equal), writing‐ review and editing (equal). **Yuxuan Huang:** resources (equal), validation (equal), writing‐review and editing (equal). **Lirong Zhou:** conceptualization (equal), validation (equal), writing‐review and editing (equal). **Xiaogang Hu:** supervision, writing‐review and editing (equal). **Xiaomin Lu:** supervision (equal), writing‐review and editing (lead). **Mingbing Xiao:** resources (lead), supervision (lead), writing‐review and editing (lead). Fei Wang, Jianshan Yang and Feng Zhu have contributed equally to this work and share first authorship. All authors have read and approved the final manuscript.

## Conflicts of Interest

The authors declare no conflicts of interest.

## Data Availability

The data that support the findings of this study are available from the corresponding author upon reasonable request.
